# Proactive Versus Reactive Control Strategies Differentially Mediate Alcohol Drinking in Male Wistars and P Rats

**DOI:** 10.1523/ENEURO.0385-23.2024

**Published:** 2024-03-25

**Authors:** M. D. Morningstar, N. M. Timme, B. Ma, E. Cornwell, T. Galbari, C. C. Lapish

**Affiliations:** ^1^Department of Psychology, Indiana University-Purdue University Indianapolis, Indianapolis, Indiana 46202; ^2^Department of Anatomy, Cell Biology, and Physiology, Stark Neurosciences, Indiana University School of Medicine, Indianapolis, Indiana 46202

**Keywords:** alcohol, cognitive control proactive reactive, in vivo extracellular electrophysiology, motivated behavior, prefrontal cortex, selected line P rat

## Abstract

Problematic alcohol consumption is associated with deficits in decision-making and alterations in prefrontal cortex neural activity likely contribute. We hypothesized that the differences in cognitive control would be evident between male Wistars and a model of genetic risk: alcohol-preferring P rats. Cognitive control is split into proactive and reactive components. Proactive control maintains goal-directed behavior independent of a stimulus, whereas reactive control elicits goal-directed behavior at the time of a stimulus. We hypothesized that Wistars would show proactive control over alcohol seeking whereas P rats would show reactive control over alcohol seeking. Neural activity was recorded from the prefrontal cortex during an alcohol seeking task with two session types. On congruent sessions, the conditioned stimulus (CS+) was on the same side as alcohol access. Incongruent sessions presented alcohol opposite the CS+. Wistars, but not P rats, made more incorrect approaches during incongruent sessions, suggesting that Wistars utilized the previously learned rule. This motivated the hypothesis that neural activity reflecting proactive control would be observable in Wistars but not P rats. While P rats showed differences in neural activity at times of alcohol access, Wistars showed differences prior to approaching the sipper. These results support our hypothesis that Wistars are more likely to engage in proactive cognitive control strategies whereas P rats are more likely to engage in reactive cognitive control strategies. Although P rats were bred to prefer alcohol, the differences in cognitive control may reflect a sequela of behaviors that mirror those in humans at risk for an AUD.

## Significance Statement

Cognitive control refers to the set of executive functions necessary for a goal-directed behavior. It is a major mediator of addictive behaviors and can be subdivided into proactive and reactive cognitive control. We observed behavioral and electrophysiological differences between outbred Wistars and the selectively bred Indiana alcohol-preferring P rats while they sought and consumed alcohol. These differences are best explained by reactive cognitive control in P rats and proactive cognitive control in Wistars.

## Introduction

Understanding how interactions between alcohol history and genotype impact cognitive function is critical for elucidating the mechanisms behind alcohol use disorder (AUD; Wilcox et al., 2014). We sought to determine if the differences between alcohol seeking in P rats and Wistars could be explained as the differences in cognitive control strategies. Cognitive control is the set of executive functions necessary for reducing uncertainty prior to an action (Mackie et al., 2013) and can be broken into dual mechanisms of proactive and reactive control (Braver, 2012). Proactive cognitive control maintains an active representation of the task at hand, whereas reactive control draws upon the representation when elicited by a cue or stimulus (Braver, 2012). Cognitive control is linked with AUD both as a risk factor and a domain that AUD disrupts (Wilcox et al., 2014; Liu et al., 2020). Refining our understanding of how subtypes of cognitive control may interact with AUD is critical. We investigated the degree to which P rats and Wistars differentially instantiate cognitive control and hypothesized that Wistars are more likely to implement proactive cognitive control, whereas P rats are more likely to implement reactive cognitive control.

The two-way conditioned access protocol (2CAP) has been previously used to assess how heritable factors result in the differences in neural activity and alcohol seeking behavior (Bell et al., 2006; McCane et al., 2014; Linsenbardt and Lapish, 2015; Linsenbardt et al., 2019; Timme et al., 2020, 2022). P rats display high alcohol seeking and consumption behaviors (Czachowski and Samson, 2002). P rats also display high levels of impulsivity (Beckwith and Czachowski, 2014; Linsenbardt et al., 2017) and impaired cognitive flexibility (De Falco et al., 2021). Increased impulsivity and decreased cognitive flexibility may be a result of decreased representations of task goals. For example, previous work has consistently shown blunted neural activity in the dorsomedial prefrontal cortex (dmPFC) prior to alcohol seeking in P, but not Wistar, rats (Linsenbardt and Lapish, 2015; Linsenbardt et al., 2019; Timme et al., 2022).

The dmPFC is critical for cognitive control (Braver, 2012). A general working model of the dmPFC function is to test for discrepancies and, if found, to modulate attentional or emotive resources to address the discrepancy (Alexander and Brown, 2011). The dmPFC must maintain a variety of stimulus–response outcomes whose representations can be thought of as task sets (Wisniewski et al., 2015; de Haan et al., 2018; Soltani and Koechlin, 2022). Task sets are neural representations critical for the rapid updating of learned behavior and assist in proactive control (Dreisbach and Haider, 2008; Dhawan et al., 2019; Soltani and Koechlin, 2022). Therefore, it would be expected that agents that engage in proactive control would exhibit strong task sets that would be reflected in an ensemble activity. These ensembles are hypothesized to fluctuate near important task features such as the cue or alcohol access. High-yield in vivo electrophysiology will allow us to determine if the changes in ensemble activity reflect the differences in cognitive control between P rats and Wistars. Dimensionality reduction techniques will be utilized to determine the most important components of the neural signal and how it changes according to changing task contingencies.

An agent that is more reactive in their alcohol seeking is not required to maintain a goal representation, and goal representations may emerge only when needed. In the 2CAP task, this would correspond to periods of alcohol access. Reactive control is correlated with impulsivity (Huang et al., 2017), and impulsivity is predictive of compulsive drinking (Finn et al., 1999; Acheson et al., 2011); therefore, it is expected that reactive control is also predicted of compulsive drinking. This may partially explain why P rats are more likely to drink through quinine-adulterated alcohol (Timme et al., 2022) given that their decisions are likely made at the time period of alcohol access, which allows little time to halt or change behavior. This further suggests that the differences in cognitive control styles are critical mediators of AUD.

We examined the differences between Wistars and P rats as they adapted to a contingency change during the alcohol seeking 2CAP task. Given that P rats are more impulsive and less flexible, we hypothesized that they would utilize reactive cognitive control strategies that do not depend upon maintained goal representations. Conversely, we hypothesized our Wistar population would utilize proactive cognitive control strategies. The hypothesized differences in cognitive control would be evident in both the behavioral and electrophysiological data. Overall, differences between each strain’s genes and drinking history should produce significant differences in how they implement reactive and proactive cognitive control.

## Materials and Methods

### Data

The present dataset was produced concurrently with Timme et al. (2022). Previous papers analyzed the effects of quinine on congruent (regular) 2CAP sessions. The present paper utilizes unpublished data from previously unexplored congruent and incongruent sessions. Congruent sessions are obtained the day prior to incongruent sessions.

### Animals

Adult, male Indiana alcohol-preferring rats (P rats) were utilized from breeding facilities at the Indiana University School of Medicine. Adult, male Wistars were acquired from Envigo (Envigo). Sixty Wistars and 23 P rats were allowed to drink in an intermittent access protocol (IAP). Following IAP, 20 Wistars and 16 P rats were selected for further 2CAP training. From those 20 Wistars and 16 P rats, 8 Wistars and 8 P rats were implanted with silicon electrodes. One Wistar had no incongruent datasets. In total, seven Wistars and eight P rats were used in the present dataset. The animals were kept on a reversed light schedule and given standard rat chow and water *ad libitum*. All the animals were single housed after arriving in our colony and throughout the experiment. All animal procedures were approved by the Indiana University–Purdue University Indianapolis School of Science Institutional Animal Care and Use Committee.

### IAP

All the animals underwent an IAP wherein they were given periodic access to 20% v/v alcohol for 2 weeks. Specifically, the animals were weighed, and then a bottle of water and a bottle of alcohol were attached to each animal’s cage for 24 h. Following 24 h, the water bottle and the alcohol bottle were weighed. The difference between the initial and final weight was obtained, and from this, the dose consumed was calculated by dividing this difference by the animal's weight (g/kg). The bottles were placed, and the animals were weighed specifically on Mondays, Wednesdays, and Fridays 2 h into the animals’ dark cycle. Following IAP, higher-drinking Wistars and lower-drinking P rats were selected to go on to the 2CAP training (Timme et al., 2022).

### Apparatus

The animals underwent the 2CAP training in a standard Med Associates shuttle box (Med Associates). All electrophysiology recordings were completed in a replica Med Associates shuttle box that allowed passage of our tethers and RGB video tracking.

### Two-way cued access protocol

**Figure 1.**
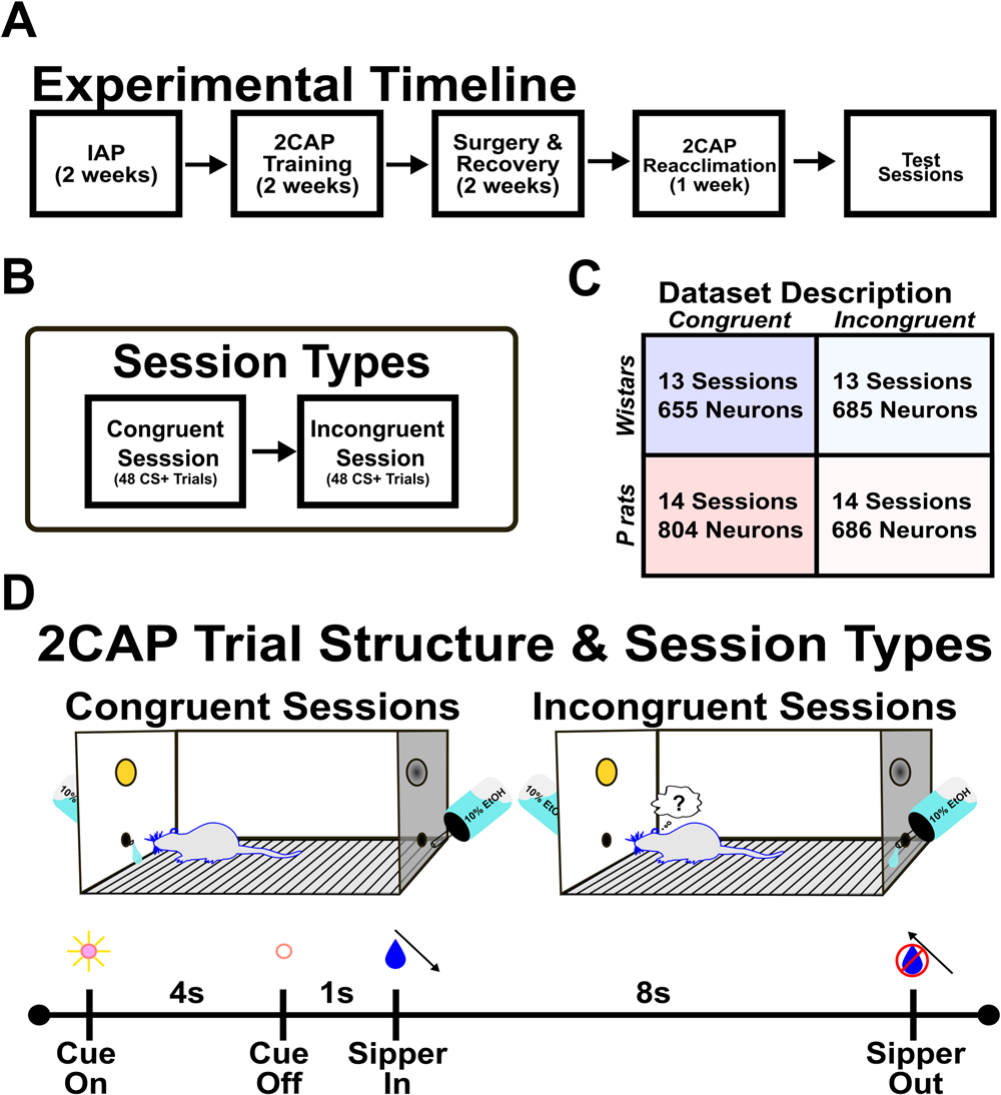
Method details and 2CAP apparatus. ***A***, Wistars and P rats underwent 2 weeks of IAP with 20% alcohol (v/v). Following IAP, Wistars and P rats were matched for consumption and underwent subsequent 2CAP training with 10% alcohol (v/v). Following the successful completion of 2CAP training, the animals underwent surgery and had silicon electrodes chronically implanted. The animals were then reacclimated to the 2CAP protocol as well as habituated to the modified 2CAP chamber. ***B***, After reacclimating, neural recordings were acquired. Each week progressed through different contingencies. We focused the present analysis on congruent sessions and incongruent sessions conducted at the end of the week. Each session type consisted of 48 CS+ trials and 48 CS− trials. ***C***, Wistars and P rats experienced roughly equal numbers of congruent and incongruent sessions. Additionally, neuron yield was relatively matched. ***D***, The 2CAP apparatus and trial structure are detailed. Briefly, during congruent sessions, alcohol access was on the same side as the CS+. During incongruent sessions, alcohol access was on the side opposite the CS+. A 2CAP trial consisted of 4 s of the CS+ being illuminated. One second after the CS+ is turned off, the alcohol-containing sipper descends. The animals then had 8 s of 10% alcohol access prior to the sipper being removed from the chamber.

The animals underwent training in the two-way cued access protocol (2CAP) for 2 weeks. The 2CAP training consisted of presentations of a conditioned stimulus (CS+) prior to the presentation of a 10% v/v alcohol sipper. An additional, separate stimulus was presented (CS−) on trials where no alcohol sipper would descend. During CS+ trials, the CS+ would illuminate on a single side of the apparatus. During normal, congruent sessions, this CS+ matched the side of the apparatus where alcohol would be present. The CS−, in contrast, was illuminated on both sides of the 2CAP apparatus. Figure 1 describes the contingency swap and apparatus. Both the CS+ and CS− were visual and could either be a solid light presented for 4 s or a blinking light (1 Hz) presented for 4 s. The type of visual stimulus was assigned randomly and counterbalanced. A single 2CAP session had 96 trials wherein 48 presented the CS+ on a random side of the apparatus and the other 48 trials presented the CS− on both sides of the apparatus. Figure 1*D* highlights the events of a single trial. During the sipper descent, both motors were active such that neither motor noise could be used as a discriminative stimulus. Following training and during electrophysiology recordings, the animals were exposed to 1–3 incongruent sessions. Incongruent sessions were when the CS+ that normally predicted the side of alcohol delivery was swapped to predict the side absent alcohol. Thus, the animals were required to adapt over a single session to the new contingency.

### Behavioral data

Behavioral data were processed with DeepLabCut (DLC; Mathis et al., 2018). Video recordings were saved in color and at 30 FPS. The training dataset identified six points in the 2CAP apparatus (each corner, both sippers) and four points on the animal’s body (snout, head cap, back, tail). Following the successful training of a neural network, all videos relevant to the current study were processed. Output data were a set of {X, Y} coordinates per identified point of interest along with the measure of confidence the neural network had for those {X, Y} coordinates at each time point. Data were preprocessed in MATLAB by interpolating the timepoints that the neural network had low confidence in (<90% log-likelihood). This ultimately allowed for an estimate of the animals’ position at each time point relative to the correct or incorrect sippers. This then permitted the quantification of correct versus incorrect sipper approaches, time spent at each sipper, and other metrics related to approach and drinking behavior with great temporal precision.

### Surgeries

Surgeries were performed on the animals following IAP and 2CAP. Presurgery 2CAP drinking was considered in the selection of which animals would move forward in the experiments such that lower-drinking P rats were matched with higher-drinking Wistars. Briefly, the animals were rendered unconscious under isoflurane, and the fur on their skull was removed. The animals were then placed in the stereotaxic frame. The animals received doses of ketoprofen (5 mg/kg, i.p.) and cefazolin (30 mg/kg, i.p.). The scalp surface was sanitized with alternating applications of betadine and 70% alcohol. Anesthetic depth was confirmed by checking muscle reflexes. Following sufficient depth of anesthesia, a topical analgesic was applied to the scalp surface (bupivacaine, 5 mg, s.c.), and then an incision was made revealing the skull. The skull surface was cleaned and cleared of all debris and tissue until bregma and lambda were visible. Four holes were drilled for anchoring screws, two holes were drilled for grounding screws, and one craniotomy was performed at the site of probe placement (3.2 mm A/P, 0.8 mm M/L, 3.0 mm D/V, 7.5° toward midline). The electrode itself was mounted on a movable microdrive that allowed for postsurgery adjustments to be made to the depth of the electrode. All portions of the microdrive and electrode that required mobility were coated in warmed antibiotic ointment prior to the application of the dental cement head cap. Following the construction of the headcap, all open connectors were taped shut, and the animal was removed from the stereotaxic equipment and placed into a recovery cage located atop a heating pad. The animal was monitored until they regained their righting reflex and then placed back into the vivarium. Postoperative health checks were done up to 7 d following the surgery.

### Electrophysiology

Implanted silicon electrodes were purchased from Cambridge Neurotech and consisted of three different models, P, F, and H, with most electrodes being of the F model. The only difference between models is the geometry of the recording sites. All electrodes came precoated in PEDOT allowing for greater biocompatibility. Omnetics (Omnetics Connector Corporation) connectors and Intan 32-channel or 64-channel headstage preamplifiers were utilized in all recordings. Intan serial peripheal interface cables were utilized to connect the headstages to our Open Ephys acquisition board (Open Ephys). Raw electrophysiological data were sampled and recorded at 30,000 Hz via an Open Ephys acquisition board. Spike sorting was done offline via Kilosort 2.0 (Pachitariu et al., 2016). Putative units were manually separated into noise, multiunit activity, or single-unit activity via Phy (https://github.com/cortex-lab/phy). From there, only single units that had fewer than 5% interspike interval (ISI) violations were considered in further analyses. Electrode placements can be found in a study by Timme et al. (2022).

### Statistical analysis

Data were assessed for normality in all cases, and either parametric or nonparametric tests were utilized afterward. All statistics were done in MATLAB 2021a (The MathWorks) using customized scripts or JASP using exported CSV files from MATLAB. Multiple comparisons were conducted where appropriate and unless otherwise stated were FDR corrected.

### Data analysis and software

#### General information

MATLAB 2021b and MATLAB 2021a were utilized to operate Kilosort 2.0 as well as process all subsequent behavioral and electrophysiological data. Python 3 was utilized to execute DLC scripts and the Phy GUI. Custom MATLAB scripts were utilized for all data processing and analysis.

#### Behavioral analyses

Data obtained and interpolated from DLC were utilized to determine the position of the animal at a temporal resolution of 0.033 ms. This allowed for precise quantification of the animals’ behaviors during each trial. Trials were either centered around the cue onset or the time of the correct approach. An approach was defined as the first timepoint when the DLC marker associated with the animal’s snout was ≤9 pixels in distance from the DLC marker associated with the sipper port. This information allowed us to quantify the latencies, correct approaches, incorrect approaches, omissions, and so forth. Correct approaches were defined as trials where the animal immediately goes to the correct, alcohol-containing sipper without checking the other port. Incorrect approaches were defined as trials where the animal visited the port where alcohol was not made available. Omissions were when the animal visited neither port during a trial. Latencies were defined as the first timepoint the animal checked the port following the sipper coming into the arena. Behavioral data were selected [−5 20] seconds from the cue onset. The probability to approach any sipper was first encoded as either a 1 or 0. From there, the probabilities were split into correct or incorrect trials. A three-trial moving average was calculated across each animal’s row of data to obtain a smoothed average. The probability to occupy the sipper was also calculated. In this analysis, a time-corresponding vector was loaded with either 1 or 0 depending on whether the animal was within 9 pixels of the sipper. Data were subsequently split into the occupancy of correct or incorrect sippers. A simple mean was then calculated across sessions to determine the time by trial average.

#### Firing rate

Data from the results of spike sorting were imported into MATLAB and transformed into a spike-timing matrix. Each column contained the spike times of an individual neuron, and each row contained the *N*th spike time. Firing rates were first binned at 100 ms. For each neuron, a Gaussian kernel was applied that considered the neurons’ average ISI and its coefficient of variation (CV) in order to set the width of the smoothing kernel on an individual basis (Lehky, 2010). The width of each neuron's smoothing kernel was determined by multiplying the square root of that neuron's mean ISI with the inverse CV.

#### Electrophysiology analyses: centered on cue onset

The first set of analyses was concerned with the neural population signals relative to the cue onset. The time window used in these analyses was from 4 s prior to the cue until 18 s after the cue [−4 18] resulting in a total of 22 s of data. This time bin was adjusted from the behavioral analyses to minimize non–task-related neural activity. Due to the animals’ decreased response over trials, only the first 15 trials were used in this analysis. This resulted in a 3D data structure with trials, time bins, and all neurons across their respective sessions, separated by strain. The mean over trials was taken for each session, and then data were concatenated. Principal component analysis (PCA) was then conducted with congruent and incongruent Wistar sessions undergoing PCA separately from congruent and incongruent P rat sessions. Figure 5 describes the data structure prior to PCA. A broken stick model was utilized to determine how many PCs to include in any subsequent analyses (Barton and David, 1956; Frontier, 1976). Briefly, each component had a theoretical variance that it must exceed to be considered relevant. The theoretical variance was calculated using Equation 1. The variance associated with each component is *b_k_*. Each component is one of the *p* total. The variable *k* corresponds to the *k*th component analyzed. For example, when *p* = 7 the first component (*k* = 1) would need to exceed 37% of the explained variance to retain:

(1) $$\begin{array}{c} b_{k}=\frac{1}{p}\overset{p}{\underset{i=k}{\sum }}\frac{1}{i}. \end{array}$$

PC projections were calculated from the mean-centered data and coefficient loadings of each condition (Wistar congruent, Wistar incongruent, P rat congruent, P rat incongruent).

#### Electrophysiology analyses: centered on approach onset

The second set of analyses was primarily concerned with the activity 2 s before and 2 s after the animals’ approach to a sipper [−2 2]. The approach time was when the animals’ snout was ≤9 pixels from the sipper port during the period of fluid access. The time window was set to allow the testing of whether strategies are encoded and implemented proactively with minimal interference from other task variables. The distance metrics for the approach were initially tested with the same PCA setup as described above. Following this, we sought to identify additional differences between approaches. We utilized the spatial nature of the 2CAP shuttle box to determine if distinct neural correlates existed for approaches to the left or right side of the box. To best compare how each strain encodes the left and right approaches, we utilized a different PCA strategy. Briefly, we grouped data by session type rather than strain. In this analysis, a mean calculated over trials was calculated for each session that contained up to 15 correct approaches for each direction (left or right). The mean of those session means was then calculated for each condition (Wistar congruent, Wistar incongruent, P rat congruent, P rat incongruent). This resulted in the left and right means for each strain and session type. Prior to PCA, the means were concatenated in time such that the first half were the timepoints associated with the left trials, and the second half were the timepoints associated with the right trials. Figure 5 details the PCA strategies used for each analysis. Following PCA, data were sorted into left and right trials. First, we utilized a PCA permutation approach. Briefly, based on the broken stick model, a set of permutations was generated using Equation 2, where *n* corresponds to the broken stick point and *k* corresponds to the desired amount of PC dimensions, which in our case was 3. Three PC dimensions were utilized for computational simplicity, it is more intuitive, and it allows for ease of comparisons across session and strain types:

(2) $$\begin{array}{c} \left(\begin{array}{c} n\\ k \end{array}\right)=\frac{n!}{k!(n-k)!}. \end{array}$$

In each permutation, 1,000 randomly selected left and right trials were compared in the 3D space. Their distance was taken at each sample. The mean distance for each permutation was then utilized as a general measure of separation for the left and right trials in that strain or session type condition. Next, a separate analysis was conducted where we analyzed raw firing rates. Neurons that were less than or greater than 0.01 or −0.01 were sorted into their relevant coefficient loading. These values gave us roughly equal proportions of positive and negative loading neurons as well as a middle, undefined proportion that was not analyzed. The raw firing rate was calculated according to each PC's positive and negative loaders. A repeated-measures ANOVA (RANOVA) was utilized to find time-by-direction interactions within the PC population. PC4 was then chosen based on both the quantitative criteria and qualitative similarity across both congruent and incongruent sessions. The mean value before and after making the approach was then calculated for each session and strain combination and those values were compared. Last, the mean session latencies were compared with the mean distance values between the left and right choices for each session. Linear regression and Pearson's correlation were calculated.

## Results

### Wistars are more likely to incorrectly approach the sipper during incongruent sessions

**Figure 2.**
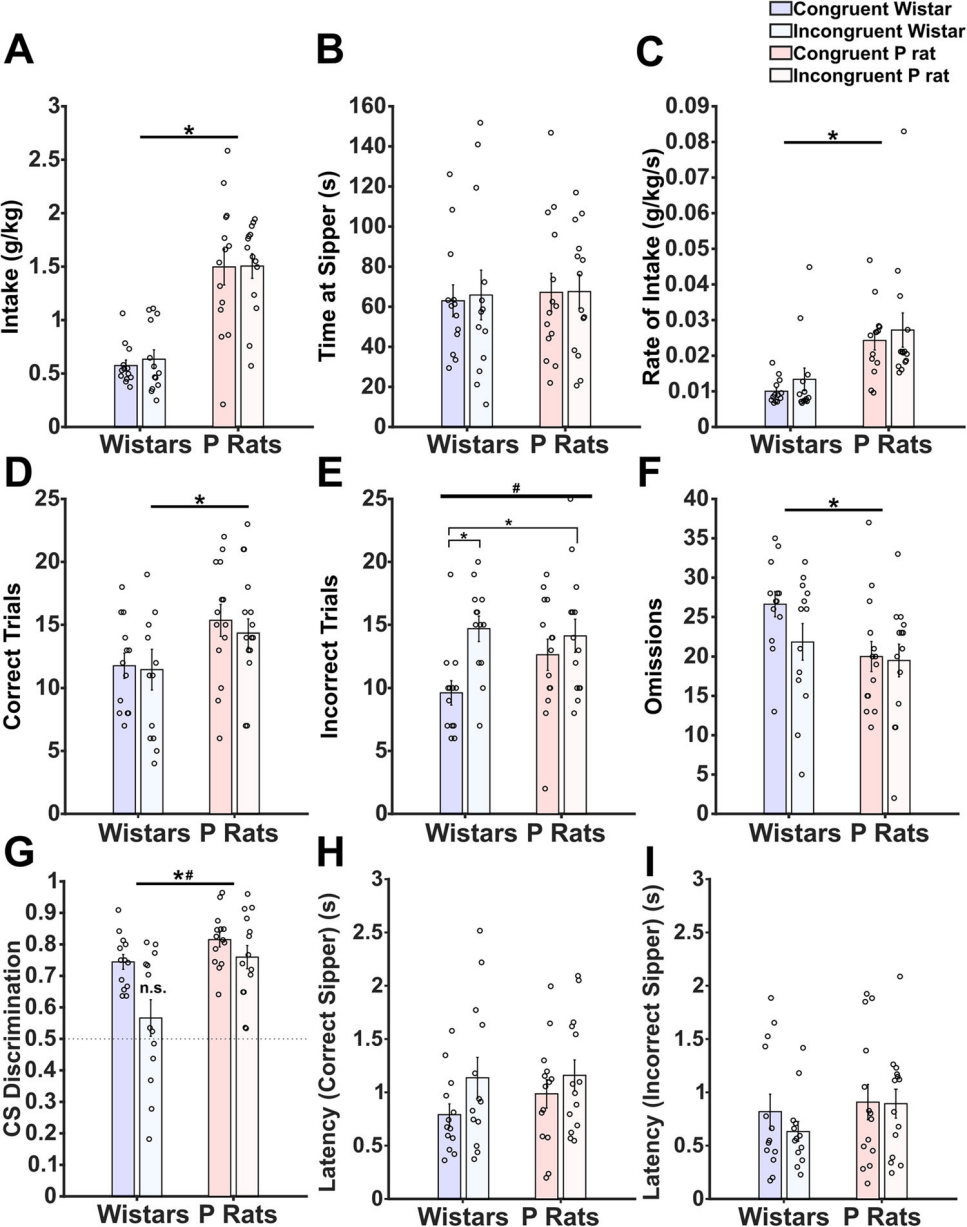
Incongruent sessions disrupt Wistars’ performance. ***A***, Intake in g/kg is shown for each strain and session type. A main effect of strain (*) was detected. ***B***, Sipper occupancy was quantified, and no main effects of either strain or session type were detected. ***C***, The rate of intake is depicted, and a main effect of strain (*) was detected. ***D***, Correct trials were determined per session, and a main effect of strain (*) was detected. ***E***, Incorrect trials were determined per session and a main effect of session type (#) was detected. Post hoc tests indicated that congruent Wistars made fewer mistakes than incongruent Wistars and incongruent P rats. ***F***, Omissions were determined per session, and a main effect of strain (*) was detected. ***G***, CS discrimination was calculated, and a main effect of strain (*) and session type (#) was detected. Additionally, the incongruent Wistars’ ratio was not different from 0.5. ***H***, Latency to approach the correct sipper is shown. A trending main effect of session type was found. ***I***, Latency to approach the incorrect sipper is shown. No main effects of strain or session type were found. A MANOVA detected significant differences between congruent and incongruent sessions in Wistars but not P rats. This suggests an overall difference in the behavioral performance exists in Wistars but not P rats when the session contingency is changed.

Wistars and P rats drank significantly different amounts throughout the task but did not show differences between session types. A main effect of strain was detected for the total alcohol intake (ANOVA, *F*_(1,50)_ = 58.8, *p* < 0.001, Fig. 2*A*). We additionally analyzed the total time at the sipper, and no main effect of strain (ANOVA, *F*_(1,50)_ = 0.093, *p* = 0.762) or session type was detected (*F*_(1,50)_ = 0.028, *p* = 0.866, Fig. 2*B*). A main effect of strain (*F*_(1,50)_ = 18.424, *p* < 0.001, Fig. 2*C*) was found for the rate of intake. Despite the changed contingency, no session type differences in drinking were observed.

While measures of alcohol consumption did not differ between session types, Wistars showed behavioral differences related to the incongruent sessions. Correct approaches are defined as approaching where the sipper has descended on the first attempt. These data show that there is a main effect of strain (ANOVA, *F*_(1,50)_ = 6.572, *p* = 0.013, Fig. 2*D*) on the number of correct approaches. Interestingly, Wistars in the incongruent sessions made an increased number of incorrect approaches. A main effect of session type was detected (*F*_(1,50)_ = 8.129, *p* = 0.006, Fig. 2*E*). Post hoc analyses indicated that congruent Wistars made significantly fewer mistakes compared with both incongruent Wistars [Tukey's honest significant difference (HSD): *p* = 0.018, 95% CI: (−5.077, −0.662)] and incongruent P rats [Tukey's HSD: *p* = 0.0375, 95% CI: (−4.528, −0.193)]. Wistars also made more omissions (ANOVA, *F*_(1,50)_ = 5.042, *p* = 0.029, Fig. 2*F*). In sum, the data indicate that incongruent trials have a modest, impairing effect on Wistars’ performance that is not detectable in P rats.

If the animal detected a change in the CS-reward contingency, this might also indicate that the meaning of the CS+/− was altered. The ratio was the amount of CS+ approaches divided by the sum of CS+ and CS− approaches. CS+ approaches included both correct and incorrect approaches. Values near 0.5 indicate that the animal approaches both CS types at equal rates. A main effect of both strain (ANOVA, *F*_(1,50)_ = 12.280, *p* < 0.001) and session type (*F*_(1,50)_ = 9.613, *p* = 0.003, Fig. 2*G*) was observed. A single sample *t* test indicated that each distribution was significantly different from 0.5 except for Wistars during incongruent sessions (*t*_(12)_ = 1.141, *p* = 0.276) suggesting that the incongruent contingency uniquely interfered with Wistars’ CS discrimination.

We additionally analyzed the latency to the sipper. The latency measure begins as soon as the sipper descends. Correct latencies required the animals to visit the correct sipper first. A trend of session type was detected (*F*_(1,50)_ = 3.18, *p* = 0.081, Fig. 2*H*) where incongruent sessions may lead to longer latencies. No main effects were observed in the latency to reach the incorrect sipper (Fig. 2*I*).

To determine the extent to which Wistars or P rats display global impairments due to incongruent sessions, we conducted a multivariate ANOVA (MANOVA) for each strain. All variables from Figure 2 were included except for the omissions due to constraints on collinearity. Overall, Wistars’ behavior was significantly altered by incongruent sessions (MANOVA, *F*_(1,24)_ = 3.452, *p* = 0.015), whereas P rats did not show global alterations due to the incongruent sessions (MANOVA, *F*_(1,26)_ = 0.557, *p* = 0.800). This suggests that Wistars are impacted more by the contingency change than P rats as evidenced by the session-wide metrics. Therefore, we hypothesized that Wistars employ proactive cognitive control that maintains the goal over time whereas P rats employ reactive cognitive control that generates a representation of the goal only when alcohol is available. To find further evidence for our hypotheses, we analyzed next the temporal aspects of Wistars’ mistakes, both across and within trials.

### Wistars make immediate mistakes during incongruent sessions

**Figure 3.**
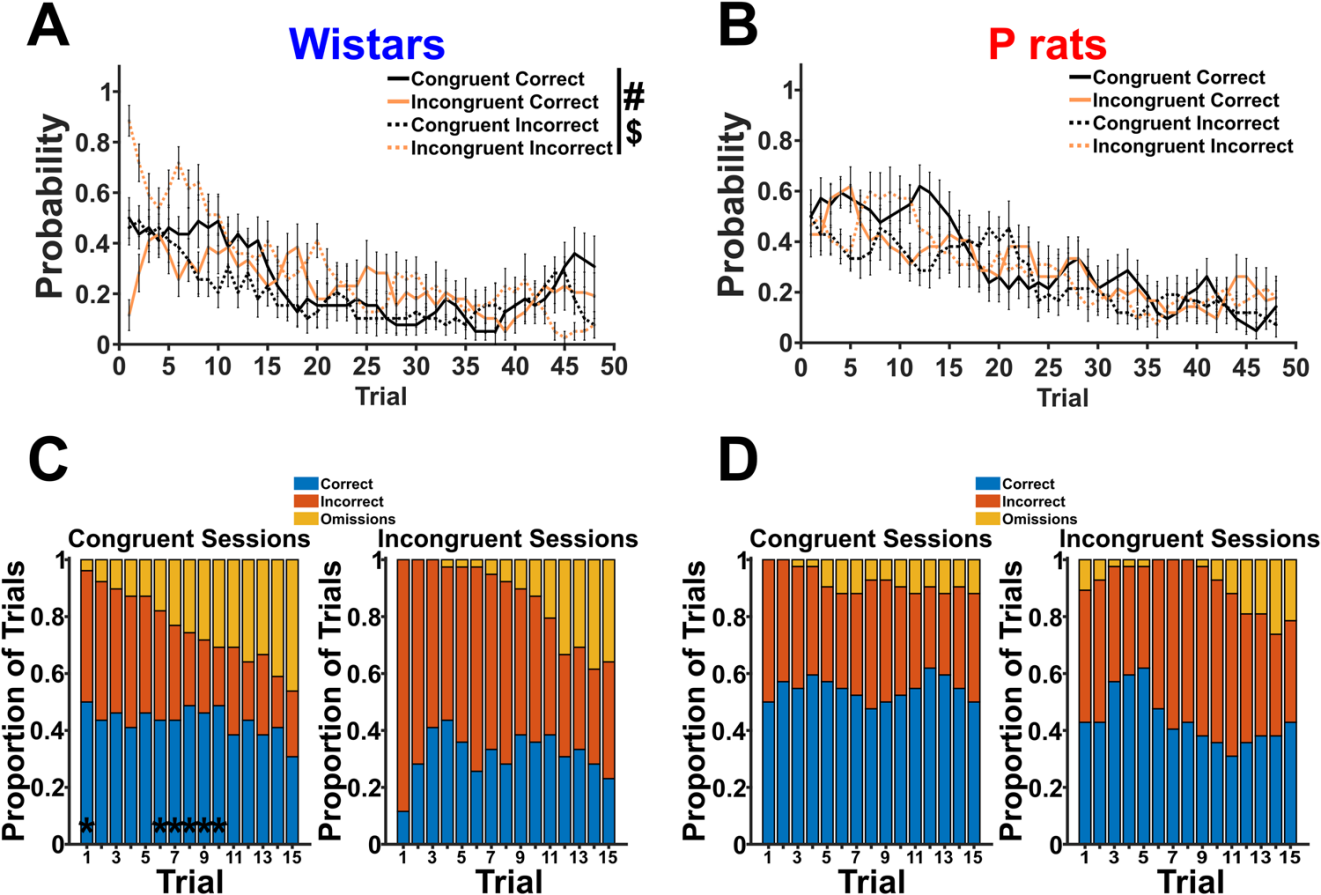
Wistars make mistakes earlier in a *session*. ***A***, The probability to make a correct or incorrect approach is shown for each session type for Wistars. A main effect of trial type ($) and session type (#) was detected. Critically, Wistars make more mistakes earlier in the session. ***B***, The same information from ***A*** is shown but for P rats. Critically, neither a main effect of trial type nor session type was detected. ***C***, The mean proportions of correct, incorrect, or omissions are shown for the first 15 trials in Wistars. The asterisks (*) denote the trials where the proportions from the incongruent sessions are significantly different than the proportions from the congruent sessions. ***D***, The same information as in ***C*** is shown but for P rats.

Wistars show deficits in the aggregate session data. We next sought to determine when within a session they show these deficits. The mean probability to approach the correct or incorrect sipper was determined for each strain and session type. Correct approaches only include those where the animal successfully approaches the alcohol-containing sipper on the first attempt. The mean probability was calculated as a three-trial moving average. In Wistars, a main effect of session type (RANOVA, *F*_(1,48)_ = 4.097, *p* = 0.0485) and an interaction between the session type and trial accuracy were observed (*F*_(1,48)_ = 5.373, *p* = 0.025, Fig. 3*A*). On early trials, Wistars exhibit decreases in correct approaches that are paralleled by an increase in incorrect approaches. This suggests that they are implementing their previously learned rule. In P rats, no between- or within-subject main effects or interactions were found. Critically, no difference in session type was observed for P rats (RANOVA, *F*_(1,52)_ = 0.056, *p* = 0.814, Fig. 3*B*). Ultimately, the data suggest that the meaningful differences in the session-wide time course of behavior in Wistars emerge as a function of the change in contingency between the CS+ and reinforcer.

Next, the difference between mean proportions of correct, incorrect, or omissions in the first 15 trials was analyzed for each strain using a chi-squared test. Asterisks on the congruent session graph denote trials where the proportions between congruent and incongruent sessions significantly differ after FDR corrections. Ultimately, we see that Wistars (Fig. 3*C*), but not P rats (Fig. 3*D*), are uniquely affected by the changed contingency early within a session.

**Figure 4.**
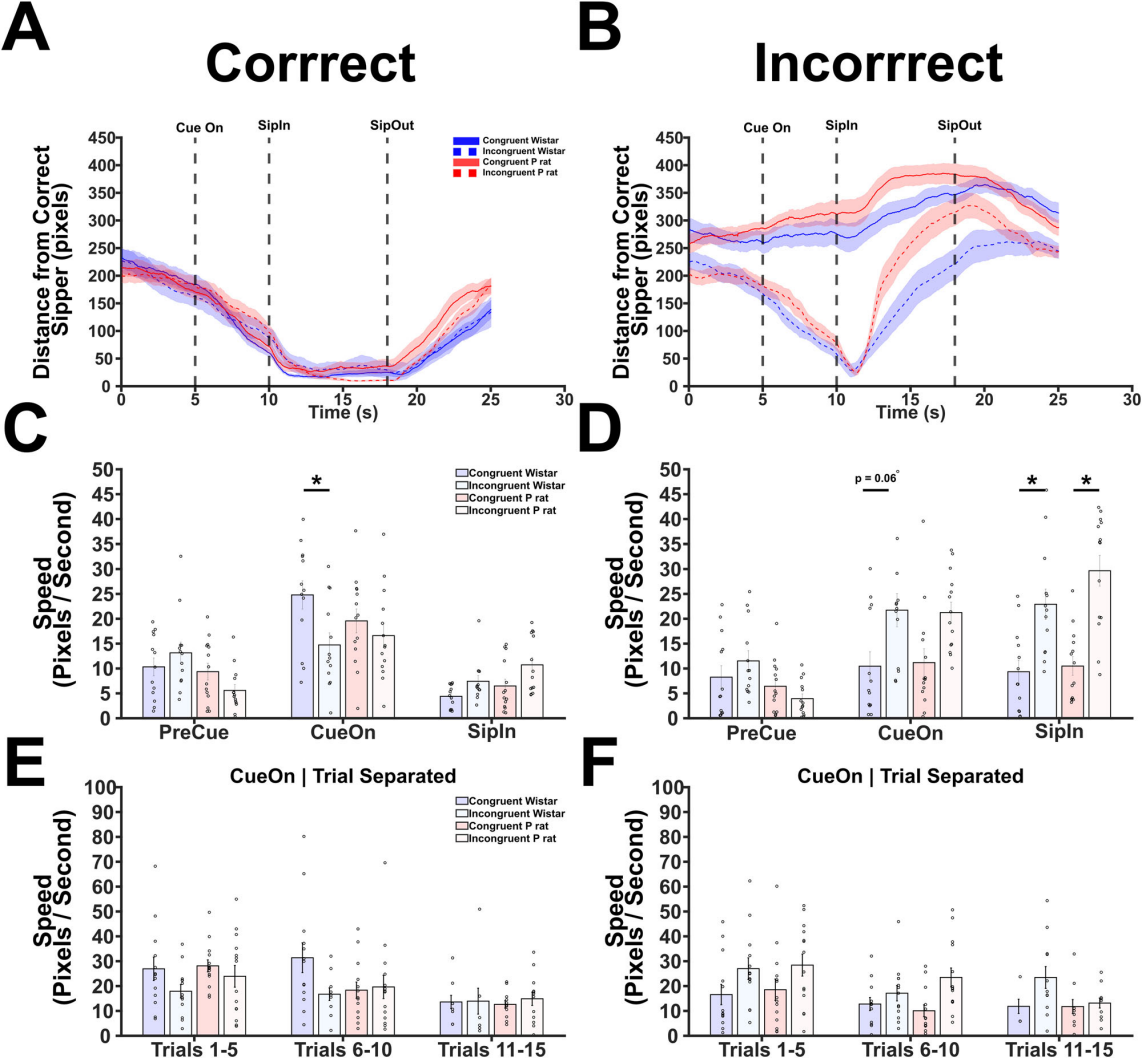
Wistars are faster to approach the correct sipper during congruent sessions and the incorrect sipper during incongruent sessions. ***A***, The distance from the correct sipper was calculated for each condition. Generally, there are no differences in the distances observed between conditions. ***B***, The distance from the incorrect sipper was calculated for each condition. The animals in incongruent sessions are likely to approach the incorrect sipper; however, P rats are faster to correct this behavior. The trial was broken down into discrete epochs. PreCue refers to the time period before the cue. CueOn refers to the time period after the cue onset but before the sipper comes in. SipIn refers to the time period after the sipper comes in and before the sipper is removed. ***C***, The speed at which the correct sipper was approached was calculated for all trial epochs. Critically, it is observed that Wistars in the congruent session move toward the correct sipper during the cue onset period faster than Wistars in the incongruent session. ***D***, The speed at which the incorrect sipper was approached was calculated for all trial epochs. During the cue onset period, Wistars in the congruent session moved slower than Wistars in the incongruent session. ***E***, The speed at which the animals moved toward the correct sipper during the cue onset period was broken down across trials. No conclusive results were observed between strain and session type or between trial blocks. ***F***, The speed at which the animals moved toward the incorrect sipper during the cue onset period was broken down across trials. Again, no differences were observed.

After finding clear evidence that Wistars are affected early within a session, we next sought to determine if measures related to the speed of a correct or incorrect approach could explain differences in session-wide behavior. Toward this, we found the distance each animal was from the correct or incorrect sipper on correct or incorrect trials. Overall, there were no differences in how each strain and condition approached the correct sipper on correct trials (Fig. 4*A*). Conversely, how the animals approached the incorrect sipper on incorrect trials exhibited several differences. First, there was a significant main effect of strain (RANOVA, *F*_(1,50)_ = 4.713, *p* = 0.0347) and session type (*F*_(1,50)_ = 92.627, *p* < 0.001). Additionally, there were significant interactions between both strain and time (*F*_(1,50)_ = 4.711, *p* = 0.0347) as well as session type and time (*F*_(1,50)_ = 68.694, *p* < 0.001, Fig. 4*B*). Next, we calculated the speed at which the animals in each condition moved during each epoch of the trial. An epoch was defined by events within the trial such as the cue onset, sipper descent, or sipper ascent. In this analysis, we focused on cue onset through the sipper inset. Speed was calculated by taking the absolute value of distance traveled during an epoch divided by the length of time of that epoch. During correct trials, the speed at which an animal moves toward the correct sipper was significantly altered by epoch (RANOVA, *F*_(2,100)_ = 48.808, *p* < 0.001). Significant interactions between epoch and condition (*F*_(2,100)_ = 3.632, *p* = 0.03) and epoch and condition and strain (*F*_(2,100)_ = 3.632, *p* = 0.003) were observed (Fig. 4*C*). Critically, we observed a significant difference between the speed at which Wistars in congruent versus incongruent sessions move at the cue onset toward the correct sipper (*t* = 3.630, *p* = 0.018). The speed at which the animals moved toward the incorrect sipper during incorrect trials saw significant main effects of epoch (RANOVA, *F*_(2,100)_ = 22.423, *p* < 0.001) and session type (*F*_(1,50)_ = 39.263, *p* < 0.001). Additionally, significant interactions were observed between epoch and strain (*F*_(2,100)_ = 3.330, *p* = 0.040) and epoch and condition (*F*_(2,100)_ = 11.632, *p* < 0.001, Fig. 4*D*). Converse to what was found during correct approaches, a trending difference between the speed at which Wistars in the congruent versus incongruent sessions move at the cue onset toward the incorrect sipper was detected (*t* = −3.225, *p* = 0.063). Interestingly, significant differences were also found during alcohol access. Specifically, congruent Wistars were slower than incongruent Wistars (*t* = −3.882, *p* = 0.008) and congruent P rats were slower than incongruent P rats (*t* = −5.695, *p* < 0.001) at this timepoint suggesting that both strains ultimately correct their behavior. We then focused our attention on whether the speed during the cue onset epoch changed over the first 15 trials. This was accomplished by finding an average speed value per animal per five-trial blocks resulting in three-trial blocks. The speed during correct approaches during correct trials was significantly affected by the trial block (RANOVA, *F*_(2,64)_ = 8.913, *p* < 0.001). Particularly, there is a decrease in speed between trial block 1 and trial block 3 (*t* = 4.219, *p* < 0.001). However, no other effects were detected (Fig. 4*E*). During incorrect trials, the speed toward the incorrect sipper during the first 15 trials was only significantly explained by the session type (RANOVA, *F*_(1,27)_ = 7.696, *p* = 0.010, Fig. 4*F*). Together, these set of analyses suggest that altering the session type produces unique, strain-dependent effects on certain time points during the task. For example, incongruent Wistars are slower moving during periods of cue onset when compared with congruent Wistars suggesting that greater deliberation is required prior to action. Critically, these effects appear relatively stable across strain and session type (Fig. 4*E,F*) suggesting that there are no major effects of acute intoxication on the animals’ ability to perform the task.

The behavioral data suggest that differences emerge between P rats and Wistars at both a baseline level and in how they solve the task when the contingency has been altered. Wistars appear to utilize a task schema that guides their behavior proactively according to the learned set of rules the task affords. When the contingency of the task changes in the incongruent sessions, they then have difficulty adapting presumedly because they are using the previously learned rule. P rats, however, solve the task through other means. These behavioral data support our overarching hypothesis that Wistars utilize proactive cognitive control whereas P rats utilize reactive cognitive control. Ultimately, the behavioral measures assessed warrant further examination. For this, high-density neural recordings were next analyzed to determine if each strain of animals differentially encoded task variables and if this then could subsequently explain the deficits in behavior observed during incongruent Wistar sessions.

### Wistars show differences in cue encoding while P rats show differences in alcohol access encoding

**Figure 5.**
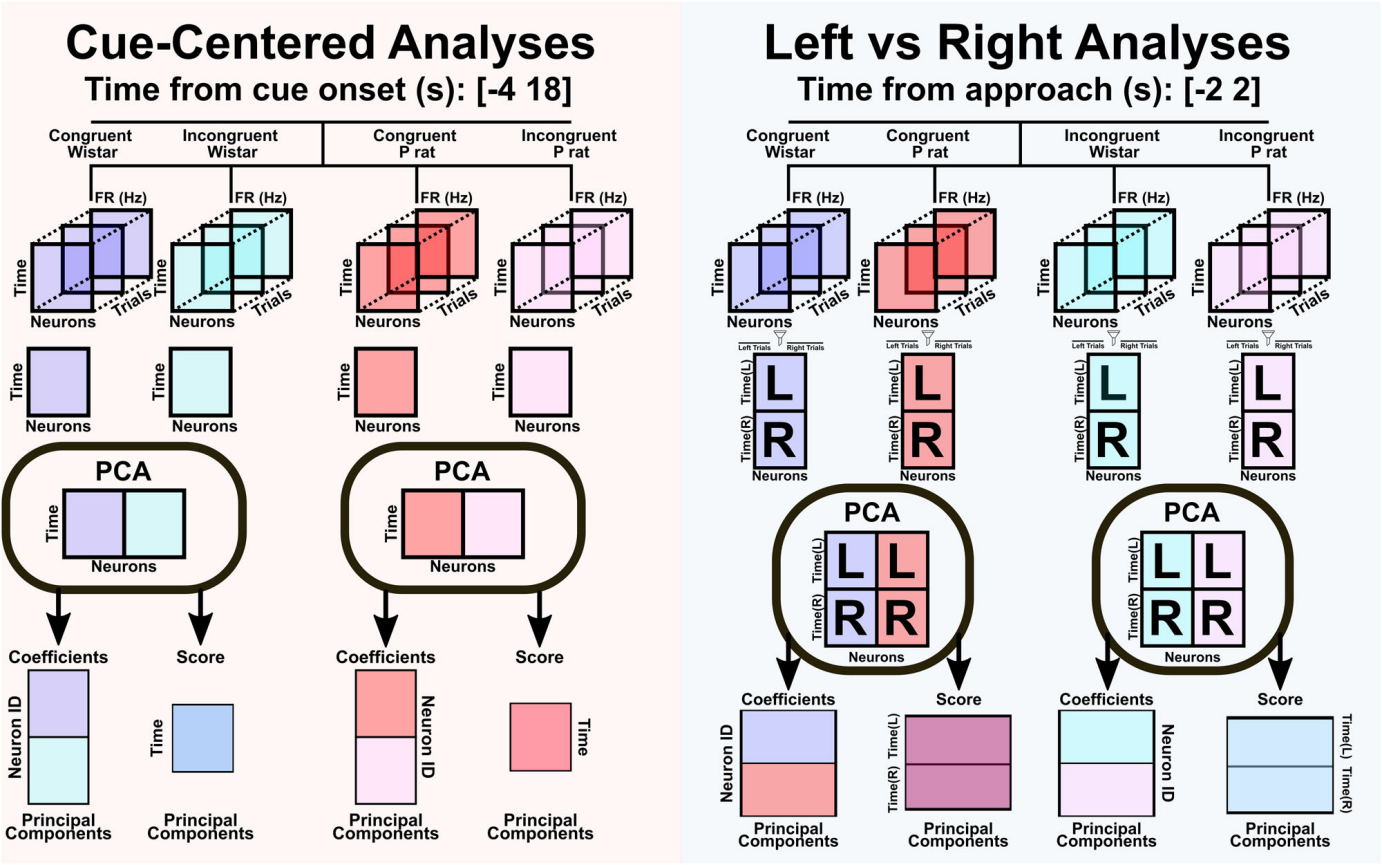
PCA methodologies for cue-centered and approach-centered analyses. Cue-centered PCA analyses were accomplished by first smoothing 100 ms binned firing rates with a Gaussian kernel. Then an NxMxT matrix was created, where *N* refers to the number of neurons, *M* refers to the number of time points, and *T* refers to the number of trials. Four seconds prior to the cue onset and 18 s after the cue onset were analyzed [−4 18] resulting in 22 total seconds. At 100 ms bins, this resulted in 221 time bins. The number of trials, *T*, was the first 15 trials of the session. The mean of trials was calculated such that an NxM matrix remained. The NxM matrix for the congruent and incongruent Wistar sessions was vertically concatenated separately from the P rat sessions. PCA was then performed, and the resulting projections and coefficients were analyzed. In the approach-centered analyses, everything was the same except that the number of time bins was greatly reduced and centered on the approach rather than the cue onset. In the left versus right analyses, up to 15 correct trials were averaged. All correct left trial means were averaged separately from all correct right trial means. Then the left and right trial means were horizontally concatenated, which doubled our M dimension. Congruent Wistar and congruent P rat sessions were then vertically concatenated. The same was done with incongruent Wistar and incongruent P rat sessions. We then ran the PCA to obtain the coefficient and score products. Following this, all PCA products were filtered into the left versus right.

**Figure 6.**
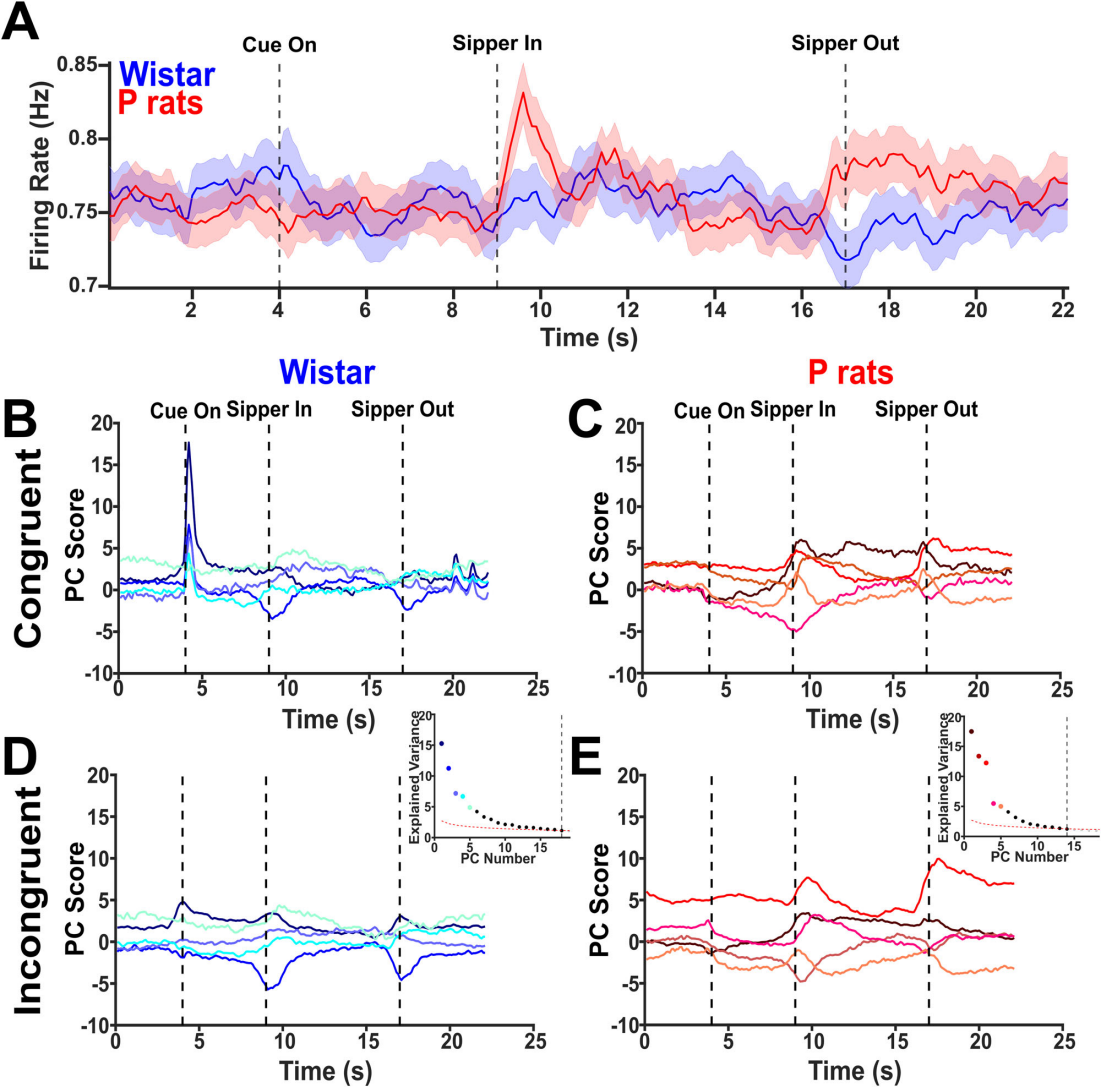
Principal components highlight several differing features between strains and session types. ***A***, The mean firing rates for each strain collapsed across session types are depicted. Further analysis of the mean firing rates can be found in Extended Data Figure 6-1. ***B***, The top 5 PCs for Wistars performing the congruent sessions are shown. Additionally, the inset describes the explained variance of each, and the dashed line indicates the broken stick point. Interestingly, several of the PCs encode the onset of the cue. ***C***, The same information as in ***B*** except for P rats is shown. Several of the PCs encode the sipper descent and ascent. ***D***, The top 5 PCs for Wistars performing the incongruent sessions are shown. Cue encoding during incongruent sessions is blunted in Wistars. ***F***, The same as in ***D*** but for P rats. PC3 shows the enhanced sipper encoding during the descent and ascent.

Figure 5 describes the data structure prior to PCA. No differences were found in the raw, averaged firing rates (Fig. 6*A*). However, a weak signal surrounding the cue onset for Wistars and strong signals surrounding the sipper descent and ascent for P rats can be qualitatively observed. Additional analysis can be found in Extended Data Figure 6-1 detailing modulatory properties of the cue and sipper on the population of neurons. PC projections of the top 5 variances explaining PCs for Wistars show that signals that were obscured in the mean become more prominent in the principal component projections. For Wistars during congruent sessions, the PC projections show large amounts of cue-locked activity (Fig. 6*B*). However, the contingency change selectively blunts this cue encoding in Wistars only (Fig. 6*D*). P rats in the congruent session intriguingly show no signals around the onset of the cue, and instead the top 5 PCs are focused on the sipper descent and ascent, which signals the start and end of alcohol availability (Fig. 6*C*). In P rats, this alcohol-onset related activity is exaggerated during incongruent sessions (Fig. 6*E*). Overall, the first 5 PCs demonstrate an overview of the neural activity in these tasks, and it indicates that neural activity in Wistars is more robustly modulated by the CS and is blunted during incongruent sessions. Conversely, P rat neural activity is robustly modulated during the periods of time corresponding to alcohol access: the sipper in and sipper out, and this is moderately altered during the incongruent session.

10.1523/ENEURO.0385-23.2024.f6-1 Figure 6-1 **Proportion and magnitude of cue and sipper modulation indicates cue and sipper are differentially expressed. A.** No differences were observed in the proportion of neurons that were either positively or negatively modulated by the cue. **B**. A main effect of strain (F (1,1317) = 4.54, p = 0.033) and an interaction of strain and session type were observed (F (1,1317) = 11.37, p < 0.001). Multiple comparisons (Tukey's HSD) indicate that neurons during incongruent P rat sessions were more strongly modulated than neurons in both congruent P rat sessions (CI: [-1.95 -0.40]) and incongruent Wistar sessions (CI: [-1.56 -0.07]). **C.** No differences were observed in the proportion of neurons that were significantly modulated by the sipper presentation. **D**. A main effect of strain (F(1,1235) = 6.870, p = 0.009) and a main effect of sign (F(1,1235) = 4.018, p = 0.045) were observed in the modulation indices. Download Figure 6-1, TIF file.

**Figure 7.**
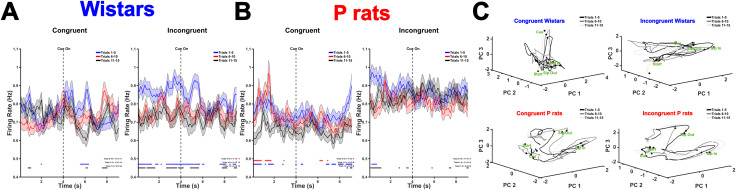
Incongruent Wistar sessions show the differential encoding of cue onset. ***A***, The mean firing rates for the full population of Wistar neurons are displayed for both congruent and incongruent sessions focused on the cue onset. During incongruent sessions, the firing rate drifts downward in later trials suggesting that Wistars are differentially encoding cue information after the contingencies are changed in incongruent sessions. The asterisks below indicate the time points where the trial blocks differed from one another. Trials 1–5 versus Trials 6–10 are indicated by black asterisks. Trials 1–5 versus Trials 11–15 are indicated by blue asterisks. Trials 6–10 versus Trials 11–15 are indicated by red asterisks. ***B***, The mean firing rates for the full population of P rat neurons are displayed for both congruent and incongruent sessions focused on the cue onset. ***C***, PC space representations for each strain and session type are depicted. Critically, the major differences observed between congruent and incongruent Wistar sessions are observed near the cue onset period suggesting that the encoding of this period is altered during incongruent sessions. These differences are not observed in P rats.

Next, raw, neural data surrounding cue onset were analyzed. From this, it was observed that Wistars in the congruent sessions showed no differences attributable to trial blocks except for sporadic periods of activity. Conversely, neural activity in incongruent Wistar sessions was significantly modulated by trial block (RANOVA, *F*_(2,1776)_ = 4.6293, *p* = 0.01), and a significant block by time interaction was observed (*F*_(2,1776)_ = 3.4884, *p* = 0.031). Neural activity that was significantly different was concentrated near the periods of cue onset (Fig. 7*A*). P rats during the congruent sessions showed a significant main effect of block (RANOVA, *F*_(2,1926)_ = 3.573, *p* = 0.0283) and a significant interaction between block and time (*F*_(2,1926)_ = 3.108, *p* = 0.045). Neural activity that was significantly different concentrated around periods of time prior to cue onset or several seconds after cue onset (Fig. 7*B*). Last, trial projections were projected into a PC space. This revealed that the PC space of congruent Wistars showed trial block invariant modulations near the cue onset whereas the PC space of incongruent Wistars was qualitatively altered. Additionally, the PC spaces of both congruent and incongruent P rats showed no major differences (Fig. 7*C*). Together, these data suggest that Wistars during incongruent sessions show differential encoding of the cue onset period as the session progresses and this corresponds with the decline in incorrect choices made.

### Wistars show left/right encoding prior to approaches, whereas P rats show left/right encoding after approaches

Next, we hypothesized that Wistars would show greater spatial encoding prior to their approaches compared with that in P rats. The differences in spatial encoding suggest that each side, left or right, has a corresponding strategy representation reflective of proactive control. Greater distances in a PC space would then suggest that each side has a well-developed internal rule guiding subsequent behavior. Dedicating more cognitive resources to differentiate these two sides suggests a greater reliance on proactive control.

**Figure 8.**
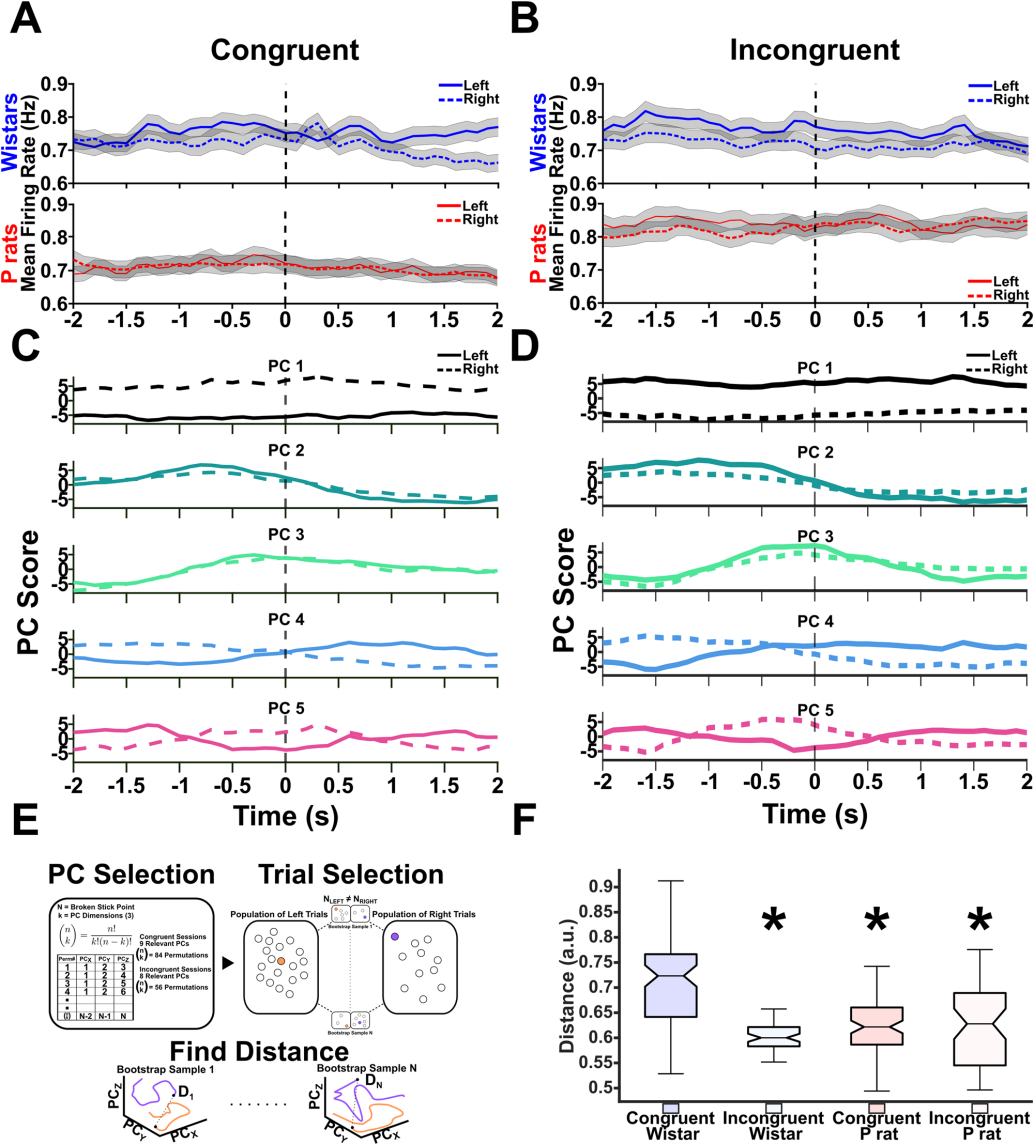
Wistars show greater spatial encoding. ***A***, ***B***, The mean firing rates for the left and right choices are depicted. ***C***, ***D***, The top 5 PCs for congruent and incongruent sessions are shown. The solid lines depict the left trials, and the dashed lines depict the right trials. ***E***, A description of the analysis performed to determine the encoding differences between the left and right trials. In brief, several permutations in a 3D PC space were identified. In each permutation, 1,000 random left and right trials were compared. The mean of those 1,000 trials was then taken for each permutation. ***F***, The results of the permutation analysis are shown. Briefly, the mean of the bootstrap samples was found for each permutation. The mean was then taken prior to approach to determine the separation of the left versus right choices prior to approach initiation. An overall effect was detected. *Tukey–Kramer post hoc tests identified that the distance between the left and right trials in the Wistar congruent session was higher than that in every other strain and session type condition.

To determine the encoding of the left versus right strategies, we organized the data differently from the first analysis prior to the PCA. First, only correct approaches were utilized where the animal successfully approached the alcohol-containing sipper on the first attempt. Next, we found all the correct approaches for the left sipper and all the correct approaches for the right sipper. Then we averaged all left approaches separate from all right approaches. In general, the raw data showed no major differences across conditions (Fig. 8*A,B*). The left and right choices were then concatenated in time such that an Nx2M matrix of the trial averaged firing rates was created, where *N* was each neuron and *M* was each timepoint. The data were then split by session type such that all congruent sessions were run together, and all incongruent sessions were run together, regardless of strain. PCA was performed on this Nx2M matrix, and the PCA products were indexed such that the left and right choices were separated once more (see Fig. 5 for an overview). Figure 8, *C* and *D*, shows the PC projections of the top 5 PCs with solid lines indicating left correct choices and dashed lines indicating right correct choices. For congruent sessions, we included the top 9 PCs, and for incongruent sessions, we included the top 8 PCs. We first sought to determine if there were any gross changes in the distance between the left and right choices. Figure 8*E* describes the data analysis process. In brief, we permuted over every possible 3D PC space for each session type. For congruent sessions, this resulted in 84 possible permutations, and for incongruent sessions, this resulted in 56 possible permutations. Within each permutation, we randomly selected 1,000 individual left and 1,000 individual right trials from the possible pool of each and calculated the PC space distance between each of these trial combinations prior to the approach time. We then took the mean of those distance combinations to determine the mean distance of each permutation. An overall group difference was observed in the distances of each condition left/right encoding [Kruskall–Wallis (3, 276), *χ*^2^ = 67.22, *p* < 0.001, Fig. 8*F*]. Tukey–Kramer multiple comparisons were used to follow up on the overall group differences. The distance between the left and right trials in the Wistar congruent sessions was significantly greater than the Wistar incongruent sessions [95% CI: (69.619, 141.393), *p* < 0.001], the P rat congruent sessions [95% CI: (40.306, 104.503), *p* < 0.001], and the P rat incongruent sessions [95% CI: (38.154, 109.929), *p* < 0.001]. No other significant differences between groups were found, including between P rat congruent and incongruent conditions. Spatial encoding is therefore higher in the Wistar congruent sessions, and, subsequently, it becomes less accurate during incongruent sessions, as evidenced by both their performance and neural encoding.

To interrogate when within a trial P rats and Wistars differentially encode the left versus right choices, we employed an additional analysis that allowed us to look at the raw firing rates of each population. In brief, neurons were indexed based on whether they were positive or negative loaders on each PC. Once again, we used a threshold of greater than/less than or equal to 0.01/−0.01. This ultimately allowed the raw, neural activity from two opposite and complementary neural populations to be compared. From this index, we pooled together neurons with the same signs from each condition and determined their means. This resulted in time-varying mean firing rates for both positive and negative loading neurons in each direction (left vs right) as well as each session type. To focus our analysis, we sought to identify PCs that selectively encoded the left and right choices and varied over the approach period. A RANOVA was conducted on the mean firing rates that were positively or negatively loaded onto each PC. PC4 solely demonstrated a significant time-by-side interaction in both the congruent and incongruent sessions (Extended Data Fig. 9-1). Qualitatively, PC4 exhibited similar firing rate characteristics in both session types.

**Figure 9.**
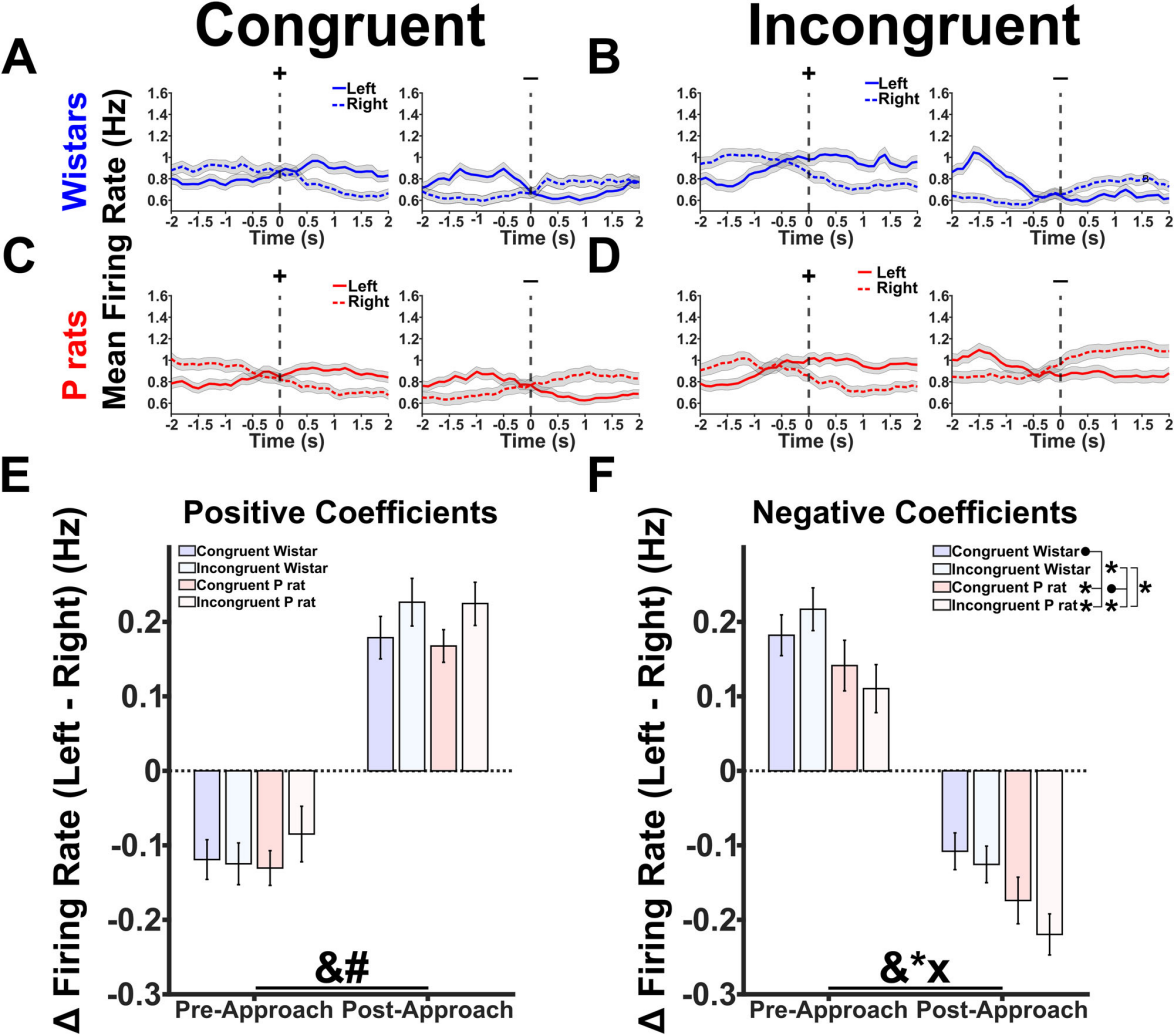
Wistars and P rats show differential spatial encoding before and after approach. Extended Data Figure 9-1 details the methodology and rationale for selecting PC4. ***A***, ***B***, The mean firing rates for neurons that either positively or negatively load onto PC4 in both the congruent and incongruent sessions for Wistars. Critically, the mean firing rate diverges at or prior to the time when each animal approaches making it a useful index of neural activity. ***C***, ***D***, The same mean firing rates for positive and negative loading neurons for PC4 in the P rat neural population. The same divergent feature at the approach time emerges. ***E***, The difference between the left and right firing rates was calculated and compared across each session type and strain combination before and after the approach for positive coefficients. A main effect of approach (&) and session type (#) was found. ***F***, The same analysis as in ***E*** but for the negative coefficients. A main effect of approach (&) and strain (*) was found. Additionally, an interaction between strain and session type was found. Further probing of this effect showed that the congruent Wistar groups were significantly different from the congruent P rat and incongruent P rat groups. The congruent P rat group was significantly different from the incongruent Wistar and incongruent P rat groups, and the incongruent Wistar group was significantly different from the incongruent P rat group.

Because of the PCs’ unique characteristics, we chose to further investigate it for Wistars in congruent and incongruent sessions (Fig. 9*A,B*) and for P rats in congruent and incongruent sessions (Fig. 9*C,D*). We were then interested in the firing rate difference between the left and right choices before and after the approach. Large firing rate differences between the left and right choices are then interpreted as an index of selective, spatial encoding. We were able to determine this by taking the difference in firing rate between each side averaging the first half prior to the approach and then averaging the second half after the approach. Positive values indicate that the mean of the condition is skewed toward encoding the left, whereas negative values indicate that the mean of the condition is skewed toward the right. A two-factor, RANOVA was used where the approach coded for whether the animal was before or after their approach. A significant main effect of approach epoch (*F*_(1,76)_ = 519.468, *p* < 0.001) and session type (*F*_(1,76)_ = 10.849, *p* = 0.002, Fig. 9*C*) was found. For negative loading neurons, an additional main effect of approach was found (*F*_(1,76)_ = 642.096, *p* < 0.001). Interestingly, a main effect of strain (*F*_(1,76)_ = 43.170, *p* < 0.001) but not session type was found. A significant interaction between strain and session type was also found (*F*_(1,76)_ = 4.021, *p* = 0.049, Fig. 9*D*). Probing this interaction shows that congruent Wistar sessions differed from congruent P rat sessions (*t*_(76)_ = 3.228, *p* = 0.006), incongruent P rat sessions (*t*_(76)_ = 5.541, *p* < 0.001), but not incongruent Wistar sessions (*t*_(76)_ = −0.523, *p* = 0.603). Congruent P rat sessions differed from incongruent P rat sessions (*t*_(76)_ = 2.313, *p* = 0.047) and incongruent Wistar sessions (*t*_(76)_ = −3.751, *p* = 0.001). Last, incongruent P rat and incongruent Wistar sessions differed from one another (*t*_(76)_ = −6.064, *p* < 0.001). Taken together, the results of Figure 9 suggest that two competing populations of neurons exist that differentially encode the left and right choices in both P rats and Wistars. The positive loading population is selective for session type, whereas the negative loading population demonstrates approach sensitivity; specifically, Wistars show greater differences in the left versus right firing rates prior to the approach, whereas P rats show greater differences after the approach. This result is in line with our previous PC space analysis and further supports our hypothesis of Wistars exhibiting proactive cognitive control whereas P rats exhibit reactive cognitive control.

10.1523/ENEURO.0385-23.2024.f9-1 Figure 9-1 **PC coefficients were utilized to analyze raw left versus right trial firing rates. A.** PC coefficients were utilized to analyze raw left versus right trial firing rates. A. PC Coefficients were split between positive (greater than or equal to 0.01) and negative (less than or equal to -0.01) coefficients. Neurons that displayed positive or negative loading to these coefficients were placed into their own respective groups. These neurons were then averaged to find the mean firing rate of each PC loading. **B.** On the left, the distribution of neurons that load onto each coefficient condition is detailed for congruent Wistar sessions. On the right, the same is shown for P rats. **C.** Raw firing rates that belonged to each coefficient condition were compared with a repeated measures ANOVA (RANOVA). PCs that had main effects are shown on the left. PCs that showed interactions between side and time are shown on the right. **D.** The same as B but for incongruent sessions. **E.** The same as C but for incongruent sessions. Download Figure 9-1, TIF file.

### Left versus right encoding distances are correlated with markers of task performance

**Figure 10.**
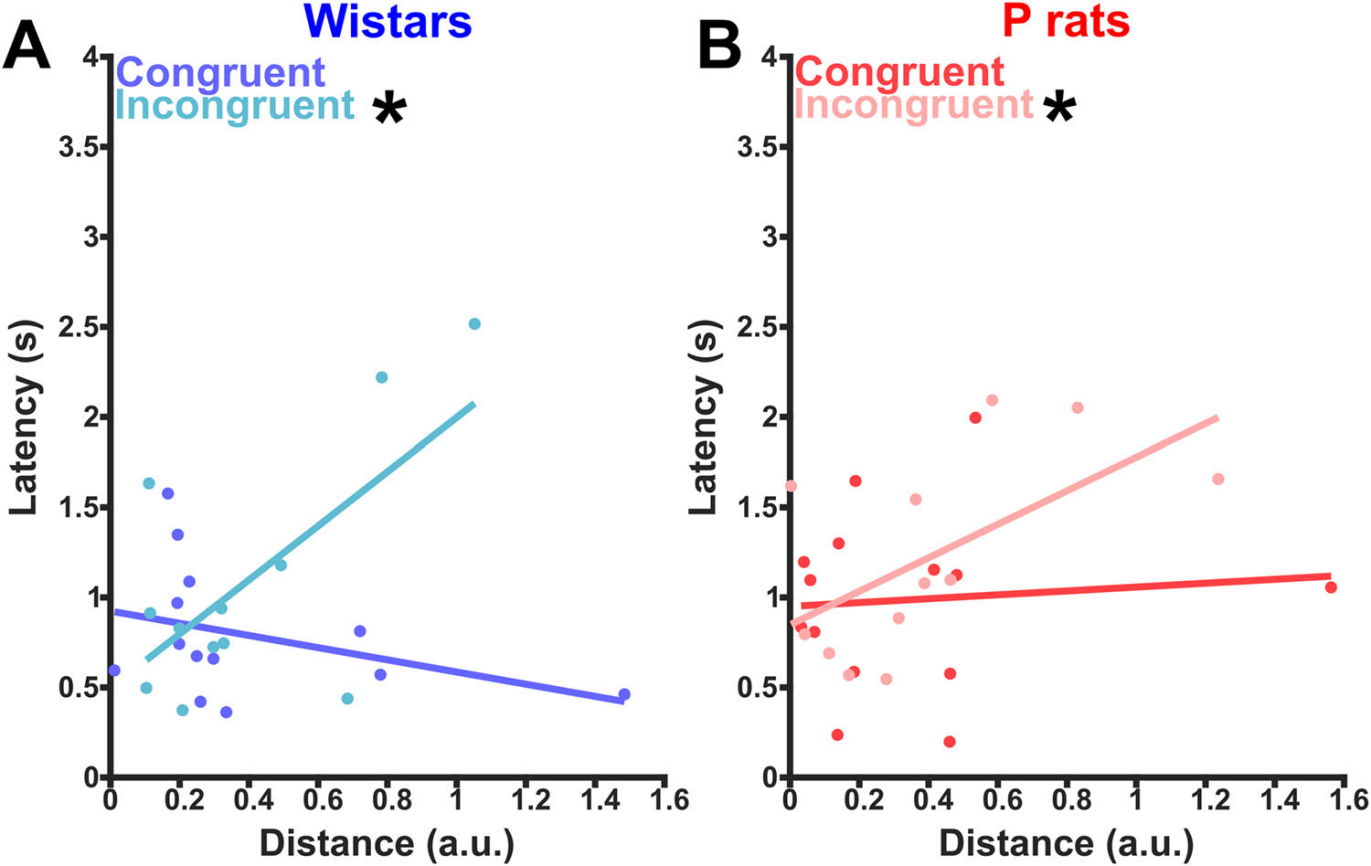
Spatial encoding is meaningfully related to latency to approach the sipper. ***A***, The correlation between latency to approach the sipper and the distance between the left and right trials for each session is shown. In the congruent condition, latency and distance are nonsignificantly and negatively correlated, whereas in the incongruent conditions, latency and distance are significantly positively correlated. Overall, Wistars show a significant interaction between distance encoding and session type. This suggests that it is beneficial to encode the side prior to moving during congruent sessions but may be detrimental during incongruent sessions. ***B***, The same information as seen in ***A*** but for P rats. In P rats, in the congruent condition, latency and distance are nonsignificantly and positively correlated, whereas in the incongruent condition, latency and distance are significantly and positively correlated.

Last, we sought to determine if the distances between the left and right trial trajectories had meaningful correlations with behavioral metrics found in Figure 2. We hypothesized that stronger representations of side information should lead to faster reaction times on congruent trials. However, if side encoding is driving behavior, this relationship should be flipped on incongruent trials with higher distances corresponding to longer reaction times. We fit a linear model to both the Wistar data and P rat data to further test if distance significantly predicted latency. For Wistars, the fit equation was as follows: Latency = 0.924–0.338 × (Distance) −0.427 × (Session) +1.839 (Distance × Session). The overall regression was statistically significant (*R*^2^ = 0.408, *F*_(1,21)_ = 4.83, *p* = 0.010). Neither distance (*β* = −0.338, *p* = 0.327) nor session (*β* = −0.427, *p* = 0.152) significantly predicted latencies. However, the interaction between distance and session was a significant predictor of latencies (*β* = 1.839, *p* = 0.004). Wistars had a significant, positive correlation only during incongruent sessions (*r* = 0.657, *p* = 0.020, Fig. 10*A*). In our linear model for P rats, the fit equation was as follows: Latency = 0.950 + 0.108 × (Distance) −0.102 × (Session) +0.822 × (Distance × Session). The overall model was not statistically significant (*R*^2^ = 0.223, *F*_(1,22)_ = 2.11, *p* = 0.128). Neither distance (*β* = 0.108, *p* = 0.759), session (*β* = −0.102, *p* = 0.723), nor interaction (*β* = 0.822, *p* = 0.147) significantly predicted latencies. Interestingly, P rats had a slight, significant positive correlation during incongruent sessions (Pearson's *r* = 0.590, *p* = 0.043, Fig. 10*B*). Taken together, these results suggest that the markers of task performance are linked with our distance metric. Specifically, our interaction suggests that it may be detrimental to encode spatial information during incongruent sessions.

## Discussion

The present data provide evidence that P rats and Wistars utilize different strategies when seeking alcohol during the 2CAP task. It was hypothesized that Wistars’ behavior is best described as being dominated by proactive cognitive control whereas P rats’ behavior is best described as being dominated by reactive cognitive control. Behavioral and neural evidence indicates that Wistars are prospectively engaged in the task while P rats tend to engage in reactive control in response to environmental stimuli. This difference in cognitive control styles may be critical as to why the two strains diverge in their drinking behaviors.

2CAP is a model of alcohol seeking in response to cues. Several studies utilizing it have been published by our lab (McCane et al., 2014; Linsenbardt and Lapish, 2015; Linsenbardt et al., 2019; Timme et al., 2020, 2022) and demonstrated strain differences between Wistars and P rats. Several of these studies have also examined the changes in the alcohol contingency whether it is through the addition of quinine (Timme et al., 2020, 2022) or the substitution of alcohol with water (Linsenbardt and Lapish, 2015; Linsenbardt et al., 2019). In both cases, P rats continue to demonstrate strong seeking behaviors despite the changes in the contingency. However, a novel angle of the present study is measuring neural activity while disrupting the contingency that predicts reward. In the aggregate, we see clear signals that Wistars are differentially affected by the changed contingencies that are not as apparent in P rats.

In the present dataset and previous work (Timme et al., 2022), we observed that the CS+ elicits approach behavior in both strains whereas the CS− does not. This is evidence that the animals have formed an association between the presentation of the CS+ and the subsequent availability of alcohol. However, as observed in the present data the specific sequence of actions the CS+ elicits likely differ between and within a strain. For at least the majority of Wistars, they can follow the cue light to the detriment of task performance in the incongruent sessions, whereas for P rats, the meaning of the CS+ seems less certain. An alternative explanation for the purpose of the CS+, particularly for animals exhibiting reactive cognitive control, may be as an occasion setter that guides attention to the more proximal cue of the noise from the sipper motors (Fraser and Holland, 2019). However, because our sipper motors are active on both sides of the 2CAP chamber, the noise is not a discriminative stimulus, which may explain why the P rats’ likelihood to make an incorrect or correct approach remains near chance in both congruent and incongruent sessions (Fig. 3*B*).

Other rodent studies have explored proactive and reactive cognitive control. Most of these have used tasks that measure perceptual decision-making and stress psychophysical measures such as reaction times. Longer reaction times often reflect reactive control processes as the agent gathers evidence (Gu et al., 2020; Hernández-Navarro et al., 2021). Shorter reaction times indicate that a task set exists, which is stimulus independent (Hernández-Navarro et al., 2021). Additionally, several parallel brain regions are involved in proactive and reactive computations such as the globus pallidus (Gu et al., 2020) and red nucleus (Brockett et al., 2020). Together, these data suggest that proactive and reactive cognitive control processes are widely distributed and competing processes. Network approaches would likely reveal differences in proactive and reactive cognitive control between P rats and Wistars.

While the current study did change the behavioral task in a way that can be considered a true “reversal,” there are points of contact with reversal tasks. Bartolo and Averbeck (2020) found that prefrontal cortex activity predicts state switches in monkeys at rates faster than what would be expected in typical reinforcement learning scenarios. Furthermore, Dalton et al. (2016) suggested that rodent dmPFC regions are critical for monitoring action–outcome contingencies, and Atilgan et al. (2022) found evidence that mice in a probabilistic reversal learning task learn the structure of the task and that dmPFC activity is related to abrupt changes in task performance, similar to the work performed by Bartolo and Averbeck (2020) as well as Durstewitz et al. (2010). Ultimately, the data suggest that the dmPFC is critical for generating task sets. Deviations from the expectancies are tracked in the dmPFC proactively, which allows for flexible and fast switching behaviors. However, specific cognitive demands are required for the dmPFC to be required for reversals (Hamilton and Brigman, 2015). Understanding how model-free or model-based learning systems interact with proactive and reactive cognitive control is a critical future direction.

The data presented herein make contact with the literature on sign and goal tracking. Sign-tracking is the tendency to attribute motivational significance to the cues that predict subsequent rewards and has been observed to mediate addictive behaviors (Flagel et al., 2009). At first glance, Wistars may appear to be sign-tracking. If Wistars were sign-tracking, we would expect them to be less sensitive to outcome devaluation (Morrison et al., 2015). However, previous research across multiple cohorts of animals suggests that they are more sensitive to outcome devaluation than P rats (Linsenbardt and Lapish, 2015; Timme et al., 2022). Additionally, if Wistars were sign-tracking, we would expect them to take longer to reach the sipper given that they may rear upward toward the CS light. However, no differences in time to the sipper were observed between genotypes. A limitation of investigating sign versus goal tracking in the present study is that large amounts of heterogeneity within a single rodent strain exist and sufficiently powered studies with both sign and goal trackers would require more animals (Flagel et al., 2009).

The conclusions presented herein are based on a literature of Wistars exhibiting greater prospective control (Linsenbardt et al., 2017) and data suggesting Wistars exhibit greater spatial selectivity (Fig. 8) as well as changes in firing across trials (Fig. 7). Together, this suggests that Wistars are drawing upon a task set when the cue is presented rather than when the sipper is presented and due to this cognitive phenotype, they make more mistakes in the beginning of an incongruent session. Articulating the differences in Wistars and P rats in the framework of cognitive control is especially important when exploring diseases such as AUD that capture heterogenous phenotypes. Understanding the differences in cognitive control in rodents will facilitate increased precision in modeling phenotypic differences in AUD.

A clear caveat of the current conclusions is that the 2CAP task was designed to assess the motivational behaviors associated with alcohol reward, but there are limitations in its utility as a cognitive task. Future work should combine psychophysical measurements with selected drinking lines to assess the differences in response latencies during tasks of perceptual decision-making. This would provide additional evidence of each strain’s cognitive control phenotype.

Cognitive control is thought to be linked with the dopamine (DA) system (Carter and Cohen, 1998; Schultz, 1998). DA has a critical role in the perception of incoming information and how those percepts, such as cues, lead to actions (Cools and d’Esposito, 2011; Jacob et al., 2013). A clinical delineation in cognitive control task performance in those with Parksinson's disease versus those with Huntington's disease suggests that prefrontal DA is critical for cognitive control (Unschuld et al., 2012; Timmer et al., 2018; Júlio et al., 2019). The differences in cortical DA may explain why P rats and Wistars differ in cognitive control. A possible mechanism for the difference between strains is the differential expression of the enzyme catechol-O-methyl transferase (COMT) gene (McCane et al., 2017). COMT has an active role in the clearance of DA from the dmPFC. COMT was observed to be underexpressed in the dmPFC of male P rats (McCane et al., 2017). While P rats generally have less extracellular DA (Engleman et al., 2006), 2CAP's cues may evoke and maintain greater cortical DA given the lack of COMT clearance, thereby invigorating reward responding and motivation to the detriment of proactive control. Additionally, the COMT inhibitor, tolcapone, was shown to suppress alcohol intake in P rats (McCane et al., 2014). Together, this suggests that the differences in the presence and clearance of DA in the dmPFC are critical for alcohol seeking phenotypes. An additional mechanism for the differences observed may be the lack of metabotropic glutamate receptor 2 (mGluR2) in P rats (Zhou et al., 2013). mGluR2 is a diffuse and inhibitory receptor (Schoepp et al., 1992; Ohishi, et al., 1993). Long-term alcohol exposure has been shown to cause decreases in mGluR2 expression in Wistars and humans (Meinhardt et al., 2013) that can be rescued through viral upregulation (Meinhardt et al., 2013). In Wistars and Long–Evans, selective activation of mGluR2 can also prevent cue-induced relapse to alcohol seeking (Bäckström and Hyytiä, 2005; Augier et al., 2016). mGluR2 receptors may mediate cognition within certain disease models by reducing cortical hyperexcitability (Gruber et al., 2010). Long-term alcohol exposure similarly enhances cortical excitability (Nimitvilai et al., 2016; Cannady et al., 2018, 2020; Hughes et al., 2020; Correas et al., 2021) suggesting that mGluR2 agonism may be a target that is capable of ameliorating differences in excitability.

Additional work is necessary to determine the baseline cognitive differences between P rats and Wistars. de Falco et al. (2021) investigated the cognitive flexibility between rodent lines in an attentional set-shifting task. It was found that P rats show deficits in flexibility that may be attributable to urgency. It is therefore critical to refine our efforts to determine how proactive or reactive cognitive control changes over the course of a 2CAP session. For instance, Stock et al. (2023) found that, in humans, acute alcohol intoxication shifted cognitive control from reactive to proactive as a compensatory mechanism. Based on this as well as the results of de Falco et al. (2021), it can be hypothesized that P rats’ proactive control may shift over the course of a session as urgency wanes, and they are forced to exert proactive control over behavior to counteract the effects of alcohol intoxication. This could be best measured with a block design that shifts contingency in the middle of the session while the animals are already intoxicated. Additionally, if sucrose controls were utilized, we would expect to see more proactive control in the middle of the session but less compared with acute alcohol intoxication.

P rats and Wistars show differences in behavioral and electrophysiological correlates of cognitive control. P rats, that preferentially use reactive control, can readily maintain similar performance in our 2CAP task following a contingency change. Wistars, that preferentially use proactive control, show more performance impairments following a contingency change. Broadly, these data suggest that the dual mechanisms framework is an important and useful approach for investigating problematic alcohol use. Simple variations in genetic background and drinking history led to profound differences in how each rodent line implemented cognitive control at both a behavioral and electrophysiological level. Determining the most important factors that influence decision-making and how they go awry during disorders such as AUD is a critical bridge between clinical and preclinical studies, and cognitive control styles are a means to readily approach these factors from the top down. Reactive and proactive cognitive control is likely a constellation of several psychological and biological factors that emerge and fluctuate according to context and needs. These are top-level, psychological phenomena that emerge from a milieu of underlying factors. Nonetheless, stable and consistent differences are observed across varying populations making them clinically useful. Improper fluctuation between control styles likely leads to aberrant decision-making that is influenced by psychological disorders and neuropathology. Determining how these fluctuations are impacted by disorders such as AUD remains an open, clinically useful question that should be further explored in concert with preclinical investigations into the physiological underpinnings.

## Synthesis

Reviewing Editor: Nathalie Ginovart, University of Geneva Decisions are customarily a result of the Reviewing Editor and the peer reviewers coming together and discussing their recommendations until a consensus is reached. When revisions are invited, a fact-based synthesis statement explaining their decision and outlining what is needed to prepare a revision will be listed below. The following reviewer(s) agreed to reveal their identity: Kurt Fraser, Ankit Sood. Note: If this manuscript was transferred from JNeurosci and a decision was made to accept the manuscript without peer review, a brief statement to this effect will instead be what is listed below.

Both reviewers expressed interest in your manuscript and agreed on the significance of the work as a novel contribution to the field. However, one reviewer raised a few questions that need to be addressed in a revision.

Please find the specific comments and queries raised by the reviewers below:

Reviewer 1:

This manuscript investigates neural encoding within the dorsal frontal cortex in rats performing a conditioned approach task where a cue is predictive of alcohol access. The authors compare neural activity in sessions where the predictive cue is presented on the same side (congruent) or opposite side (incongruent) of where the sipper will be presented. The authors make use of a number of excellent analyses to identify neural mechanisms underlying this behavior. As an additional layer the authors contrast recordings between "wild-type" Wistar rats, a common inbred rat strain that happens to exhibit low alcohol drinking, and a genetically inbred derivative of this strain selected for alcohol drinking, P rats. There are minor behavioral differences between these rat strains in this task - mainly the P rats seems to perform better in incongruent sessions overall because they are less influenced by the cue than Wistar rats. The authors reveal that the population dynamics of frontal cortical activity are different between the rat strains and they suggest that this difference underlies the behavioral differences among strains. The authors have been highly responsive to the previous round of review. I have some additional comments that they may wish to consider.

1) The link between behavior in this task and two arbitrarily distinct forms of cognitive control is tedious. This has not been well-addressed in this revision by the authors given that the evidence for this is just not strongly supported by the behavior of the animals in their task. There is no reason that this pro- vs. re-active framework fits the data better than say, sign-tracking to the cue causing behavioral deficits (particularly in pigeons when the cue is presented above or away from a food hopper, Hearst & Jenkins 1974; this task was reminiscent of negative automaintenance to this reviewer). Or it could be a tendency to attribute more value to the goal than to the cue, or a sensitivity to more proximal or distal signals of alcohol availability. Neither of these alternative, equally likely, potential mechanisms are considered. The introduction gives the impression that because they are recording in the dorsal frontal cortex there is reverse inference about what must fall out of the neural data conceptually. The evidence behaviorally that it is truly a difference in cue processing versus waiting for the sipper to be presented is not very strong (there is no evident dichotomy in behavior - if anything in Wistar rats there appears to be a separation between two groups of rats who learn the incongruent task faster?). The new plots in Figure 4 suggest that the behavioral trajectories of these two rat strains are essentially identical - so how could the argument that they engage completely disparate cognitive strategies be supported?

2) There is little evidence presented to demonstrate that under baseline conditions the animals in either group understood the cue-sipper location contingency. Why is the discrimination so low in this task? Were there differences in acquisition for either group? It is concerning that at baseline the number of "correct" and "incorrect" trials is similar.

3) The schematics in J and K of Figure 2 are not necessary nor supportive of the data presented following this figure.

4) I felt the authors could have better addressed Original Reviewer 1 Point 1 better. There is a subset of rats in either strain that perform similarly (see the apparent median split in Wistars in Fig 2G). If the authors consider only neurons from these sets of comparable rats do they still see differences in their neural encoding?

5) Lines 553-554 - at baseline outside of tasks the rat strains drink less so there is no data presented here to explain that these strategies proposed explain their drinking? P rats drink more than Wistars regardless of the behavioral session?

6) Can the authors provide recording tracks to rule out that differences in density of neurons in different dorsal frontal cortex subregions can explain the results?

7) For the analysis in Figure 6A, the authors present the firing rate of all neurons summed together if I understand this correctly. If the authors were to take a more "traditional" approach, are there differences in the proportion or strength of significant cue- versus -sipper modulated neurons?

Reviewer 2:

The manuscript compares behavior and neural responses from the dmPFC of Wistar and P rats during a cue-related alcohol-seeking task. The authors found that Wistar rats made more mistakes on incongruent sessions than P rats indicating a lack of flexibility in their approach to the changed contingency. The authors then analyzed neural responses from the two groups while they were performing the task. They found differences in neuronal activity within the dmPFC that suggest that the two groups represent different elements of the task differentially and that potentially leads to a difference in the behavioral measures. The experiments are well designed and the statistical analysis is comprehensive. The paper adds to existing knowledge about alcohol-seeking behavior and suggests that individuals susceptible to developing alcohol use disorder may process alcohol-related cues differently within their mPFC than individuals that are not vulnerable.

Minor points:

1. since the authors did not use female rats in the study, the title and abstract should clearly mention that the findings are from male rats.

2. Fig 4- spelling of 'correct' and 'incorrect'.

## Author Response

Synthesis Statement for Author (Required):

Both reviewers expressed interest in your manuscript and agreed on the significance of the work as a novel contribution to the field. However, one reviewer raised a few questions that need to be addressed in a revision.

Please find the specific comments and queries raised by the reviewers below:

Reviewer 1:

This manuscript investigates neural encoding within the dorsal frontal cortex in rats performing a conditioned approach task where a cue is predictive of alcohol access. The authors compare neural activity in sessions where the predictive cue is presented on the same side (congruent) or opposite side (incongruent) of where the sipper will be presented. The authors make use of a number of excellent analyses to identify neural mechanisms underlying this behavior. As an additional layer the authors contrast recordings between "wild-type" Wistar rats, a common inbred rat strain that happens to exhibit low alcohol drinking, and a genetically inbred derivative of this strain selected for alcohol drinking, P rats. There are minor behavioral differences between these rat strains in this task - mainly the P rats seems to perform better in incongruent sessions overall because they are less influenced by the cue than Wistar rats. The authors reveal that the population dynamics of frontal cortical activity are different between the rat strains and they suggest that this difference underlies the behavioral differences among strains. The authors have been highly responsive to the previous round of review. I have some additional comments that they may wish to consider.

We thank the reviewer for their thoughtful analysis of the present work.

1) The link between behavior in this task and two arbitrarily distinct forms of cognitive control is tedious. This has not been well-addressed in this revision by the authors given that the evidence for this is just not strongly supported by the behavior of the animals in their task. There is no reason that this pro- vs. re-active framework fits the data better than say, sign-tracking to the cue causing behavioral deficits (particularly in pigeons when the cue is presented above or away from a food hopper, Hearst & Jenkins 1974; this task was reminiscent of negative automaintenance to this reviewer). Or it could be a tendency to attribute more value to the goal than to the cue, or a sensitivity to more proximal or distal signals of alcohol availability. Neither of these alternative, equally likely, potential mechanisms are considered. The introduction gives the impression that because they are recording in the dorsal frontal cortex there is reverse inference about what must fall out of the neural data conceptually. The evidence behaviorally that it is truly a difference in cue processing versus waiting for the sipper to be presented is not very strong (there is no evident dichotomy in behavior - if anything in Wistar rats there appears to be a separation between two groups of rats who learn the incongruent task faster?). The new plots in Figure 4 suggest that the behavioral trajectories of these two rat strains are essentially identical - so how could the argument that they engage completely disparate cognitive strategies be supported? We appreciate the careful examination of our present work. We agree that we did not provide adequate contextualization for why we believe differences in cognitive control strategies are the best explanation and we have provided this information in the discussion (Line 603-629). In our discussion, we have included our best argument for and against the present explanation and compared it with the phenomena mentioned above. Thank you for this suggestion, we believe it has greatly added to the scholarship of our work.

In addition, Figure 4 contains many differences that are unique to approaches towards the incorrect sipper, and it is those subtle differences in the approach to the incorrect sipper that are critical for the present paper. For instance, the trajectories to the incorrect sipper show significant main effects of strain and session type as well as interactions between strain and time as well as session type and time. Detailed statistics can be found in lines 354 - 397.

2) There is little evidence presented to demonstrate that under baseline conditions the animals in either group understood the cue-sipper location contingency. Why is the discrimination so low in this task? Were there differences in acquisition for either group? It is concerning that at baseline the number of "correct" and "incorrect" trials is similar.

Within the alcohol literature, there is heterogeneity in when an animal chooses to drink. Some animals exhibit 'front-loading' behaviors while others approach a uniform drinking distribution over time. Given that performance in the task is inextricably linked to drinking motivation, the similarity in the correct versus incorrect probability to approach the sipper is in part driven by differences in consummatory behaviors. When aggregated to a mean, if half the animals choose to drink on a trial and the other half do not, the mean probability approaches 0.5. Given that the animals are not food or water deprived and that the animals have plenty of trials to approach their preferred level of intoxication, variations in trial-by-trial motivation to consume alcohol is likely the explanation. A second explanation is that in this task, there is no punishment other than a reduction in sipper access time for initially wrong choices. The animals can simply choose to go to the opposite end. In these cases, that animal would still be making an incorrect choice according to the definition. There are pros and cons to how open-ended our drinking task is, and unfortunately one of the cons is the lack of homogenous responding.

3) The schematics in J and K of Figure 2 are not necessary nor supportive of the data presented following this figure.

The schematics have been removed.

4) I felt the authors could have better addressed Original Reviewer 1 Point 1 better. There is a subset of rats in either strain that perform similarly (see the apparent median split in Wistars in Fig 2G). If the authors consider only neurons from these sets of comparable rats do they still see differences in their neural encoding? To determine of two modes exist in these data bimodality was assessed in this subset of data using Hartigan's Dip Test. A Dip statistic of p = 0.0976 was obtained indicating that the values within are not bimodal. In the future, more animals will be added to this task thus allowing this excellent question to be assessed with sufficient precision.

5) Lines 553-554 - at baseline outside of tasks the rat strains drink less so there is no data presented here to explain that these strategies proposed explain their drinking? P rats drink more than Wistars regardless of the behavioral session? In general, P rats do drink more than Wistars regardless of behavioral session. Efforts were made to control for drinking history at the onset of 2CAP task training (see supplementary of Timme, N.M., Ma, B., Linsenbardt, D. et al. Compulsive alcohol drinking in rodents is associated with altered representations of behavioral control and seeking in dorsal medial prefrontal cortex. Nat Commun 13, 3990 (2022). https://doi.org/10.1038/s41467-022-31731-4). However, over time the distributions settled to what is seen in in Figure 2A. Animals in each session reach their desired level of intoxication and then generally proceed to omit trials. Additionally, these consumption values are stable over sessions as evidenced by similar values in the above paper despite completely different sessions being analyzed (Figure 1E of Timme et al., 2022) and are only changed by altering the taste of alcohol rather than any cues that predict alcohol's presence. Ultimately, while the magnitude of their drinking is quite different, the relative motivation to drink remains constant across our sessions.

6) Can the authors provide recording tracks to rule out that differences in density of neurons in different dorsal frontal cortex subregions can explain the results? Recording tracks have been previously published in Supplemental Figure 19 of the following paper: Timme, N.M., Ma, B., Linsenbardt, D. et al. Compulsive alcohol drinking in rodents is associated with altered representations of behavioral control and seeking in dorsal medial prefrontal cortex. Nat Commun 13, 3990 (2022). https://doi.org/10.1038/s41467-022-31731-4. Silicon electrodes were mounted on movable microdrives that spanned from the ventral ACC / dorsal PL to the ventral PL / dorsal IL. Silicon electrodes were lowered each week. Generally, the authors expect the probes to be in the mid-to-ventral PL by the time of these recordings. Additionally, all comparisons between Congruent and Incongruent sessions within a subject would be within that same population of neurons given that they occurred within the same week.

7) For the analysis in Figure 6A, the authors present the firing rate of all neurons summed together if I understand this correctly. If the authors were to take a more "traditional" approach, are there differences in the proportion or strength of significant cue- versus -sipper modulated neurons? The initial observation is correct. We have provided additional analyses to examine this comment. Briefly, a paired t-test was conducted on each neuron. 2-seconds prior to the cue or sipper and 2-seconds after the cue or sipper were examined. FDR corrections for multiple comparisons were conducted to avoid alpha inflation. The remaining proportion of neurons was examined as well as the strength via each neuron's t-statistic. Only significant t-statistics were subsequently evaluated. No differences in the proportions of neurons significantly modulated by each event were observed. A main effect of strain and a strain by session type interaction were observed for cue-modulated neurons wherein the interaction was driven by higher modulation in incongruent P rats compared with congruent P rats as well as incongruent Wistars. A main effect of strain and a main effect of sign were observed for sipper-modulated neurons. Together this indicates subtle but noticeable effects differentially expressed in cue versus sipper modulated neurons which agrees with other results of the paper.

Reviewer 2:

The manuscript compares behavior and neural responses from the dmPFC of Wistar and P rats during a cue-related alcohol-seeking task. The authors found that Wistar rats made more mistakes on incongruent sessions than P rats indicating a lack of flexibility in their approach to the changed contingency. The authors then analyzed neural responses from the two groups while they were performing the task. They found differences in neuronal activity within the dmPFC that suggest that the two groups represent different elements of the task differentially and that potentially leads to a difference in the behavioral measures. The experiments are well designed and the statistical analysis is comprehensive. The paper adds to existing knowledge about alcohol-seeking behavior and suggests that individuals susceptible to developing alcohol use disorder may process alcohol-related cues differently within their mPFC than individuals that are not vulnerable.

Minor points:

1. since the authors did not use female rats in the study, the title and abstract should clearly mention that the findings are from male rats.

The title has been updated.

2. Fig 4- spelling of 'correct' and 'incorrect'.

Thank you for pointing out this error, the spellings have been corrected from 'Corrrect' to 'Correct' etc.
