# Supplementary figures and images for: Proactive Versus Reactive Control Strategies Differentially Mediate Alcohol Drinking in Male Wistars and P Rats
Source: eNeuro. 2024 Mar 25;11(3):ENEURO.0385-23.2024. doi: 10.1523/ENEURO.0385-23.2024 (PMC10972740; doi:10.1523/ENEURO.0385-23.2024)

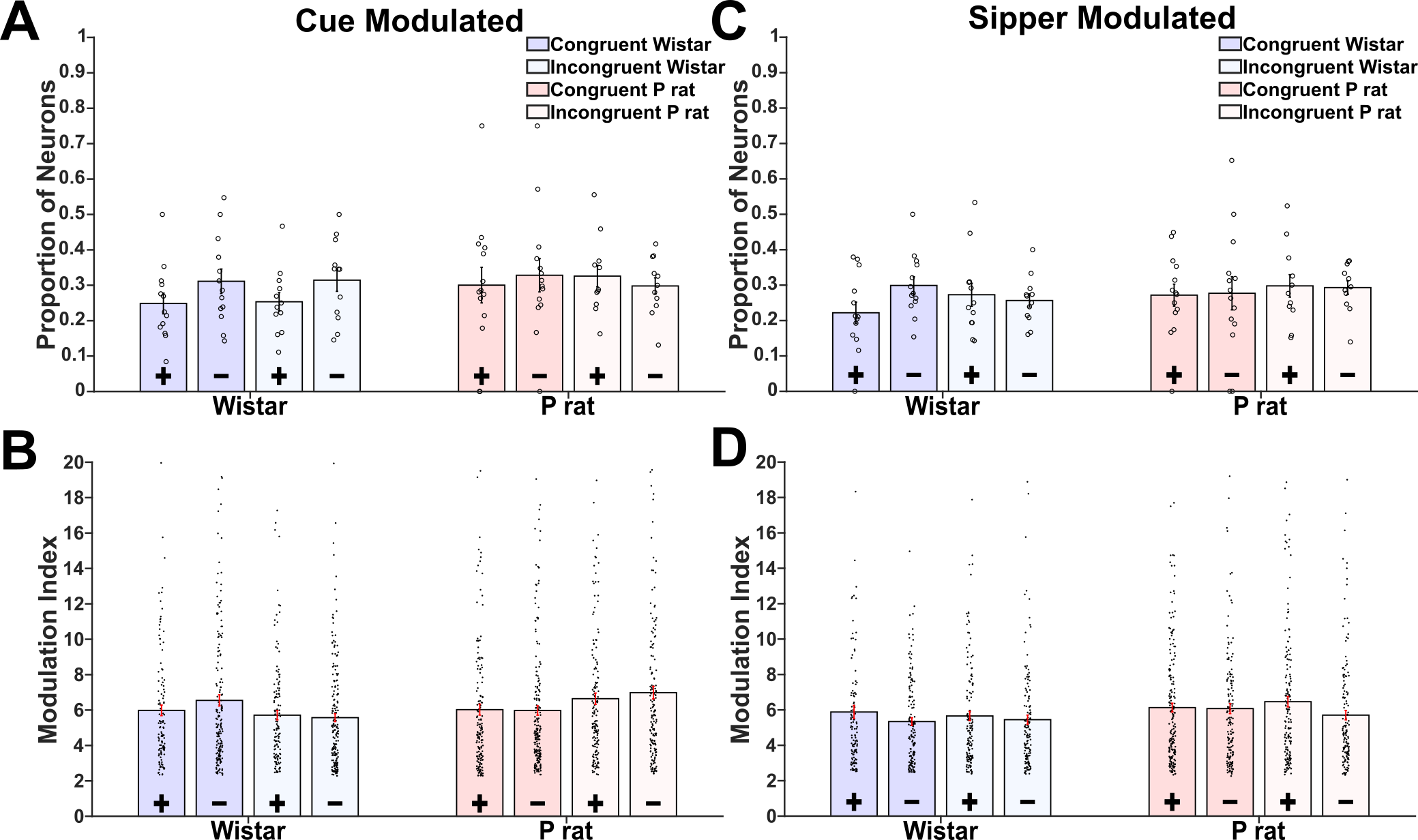

Supplement: Figure 6-1 — Proportion and magnitude of cue and sipper modulation indicates cue and sipper are differentially expressed. A. No differences were observed in the proportion of neurons that were either positively or negatively modulated by the cue. B. A main effect of strain (F (1,1317) = 4.54, p = 0.033) and an interaction of strain and session type were observed (F (1,1317) = 11.37, p < 0.001). Multiple comparisons (Tukey's HSD) indicate that neurons during incongruent P rat sessions were more strongly modulated than neurons in both congruent P rat sessions (CI: [-1.95 -0.40]) and incongruent Wistar sessions (CI: [-1.56 -0.07]). C. No differences were observed in the proportion of neurons that were significantly modulated by the sipper presentation. D. A main effect of strain (F(1,1235) = 6.870, p = 0.009) and a main effect of sign (F(1,1235) = 4.018, p = 0.045) were observed in the modulation indices. Download Figure 6-1, TIF file. [file eneuro-11-ENEURO.0385-23.2024-s001.tif]

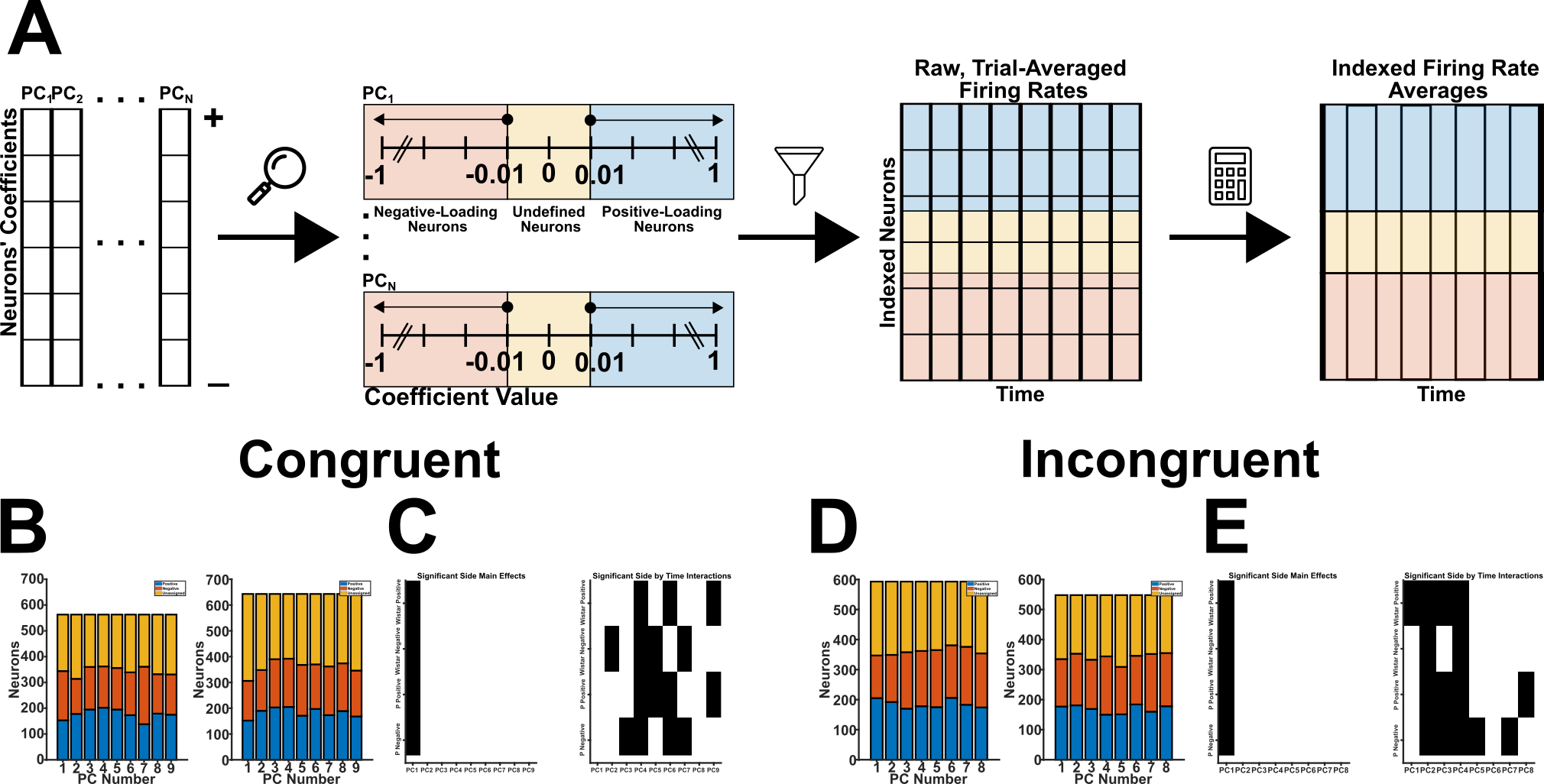

Supplement: Figure 9-1 — PC coefficients were utilized to analyze raw left versus right trial firing rates. A. PC coefficients were utilized to analyze raw left versus right trial firing rates. A. PC Coefficients were split between positive (greater than or equal to 0.01) and negative (less than or equal to -0.01) coefficients. Neurons that displayed positive or negative loading to these coefficients were placed into their own respective groups. These neurons were then averaged to find the mean firing rate of each PC loading. B. On the left, the distribution of neurons that load onto each coefficient condition is detailed for congruent Wistar sessions. On the right, the same is shown for P rats. C. Raw firing rates that belonged to each coefficient condition were compared with a repeated measures ANOVA (RANOVA). PCs that had main effects are shown on the left. PCs that showed interactions between side and time are shown on the right. D. The same as B but for incongruent sessions. E. The same as C but for incongruent sessions. Download Figure 9-1, TIF file. [file eneuro-11-ENEURO.0385-23.2024-s002.tif]
